# GFI1-driven transcriptional and epigenetic programs maintain CD8^+^ T cell stemness and persistence

**DOI:** 10.1038/s41590-025-02151-5

**Published:** 2025-05-15

**Authors:** M. Zeeshan Chaudhry, Evelyn Chen, Hiu On Man, Aneesha Jones, Renae Denman, Huiyang Yu, Qiutong Huang, Adrian Ilich, Jaring Schreuder, Severine Navarro, Zewen K. Tuong, Gabrielle T. Belz

**Affiliations:** 1 The University of Queensland Frazer Institute, University of Queensland, Woolloongabba, Queensland Australia; 2 Ian Frazer Centre for Children’s Immunotherapy Research, Child Health Research Centre, The University of Queensland, Woolloongabba, Queensland Australia; 3 QIMR Berghofer Medical Research, Herston, Brisbane, Queensland Australia

**Keywords:** Immunological memory, Lymphocyte differentiation

## Abstract

Long-lived memory CD8^+^ T cells are essential for the control of persistent viral infections. The mechanisms that preserve memory cells are poorly understood. Fate mapping of the transcriptional repressor GFI1 identified that GFI1 was differentially regulated in virus-specific CD8^+^ T cells and was selectively expressed in stem cell memory and central memory cells. Deletion of GFI1 led to reduced proliferation and progressive loss of memory T cells, which in turn resulted in failure to maintain antigen-specific CD8^+^ T cell populations following infection with chronic lymphocytic choriomeningitis virus or murine cytomegalovirus. Ablation of GFI1 resulted in downregulation of the transcription factors EOMES and BCL-2 in memory CD8^+^ T cells. Ectopic expression of EOMES rescued the expression of BCL-2, but the persistence of memory CD8^+^ T cells was only partially rescued. These findings highlight the critical role of GFI1 in the long-term maintenance of memory CD8^+^ T cells in persistent infections by sustaining their proliferative potential.

## Abstract (web summary)

Belz and colleagues show that GFI1, a transcriptional repressor that recruits histone-modifying enzymes, is necessary for the generation and maintenance of stem cell memory CD8^+^ T cells.

## Main

Antigen recognition by naive CD8^+^ T (T_N_) cells initiates clonal expansion and a hierarchical differentiation program that generates heterogeneous populations, including short-lived effector CD8^+^ T (T_SLEC_) cells with potent cytotoxicity and memory CD8^+^ T (T_M_) cells that confer durable immunity. CD8^+^ T_M_ cells can persist for decades, even without re-exposure to cognate antigens^1–3^. These long-term responses are sustained by central memory CD8^+^ T (T_CM_) cells and stem cell memory CD8^+^ T (T_SCM_) cells that retain both self-renewal and immune reconstitution potential^4,5^. CD8^+^ T_CM_ cells and CD8^+^ T_SCM_ cells emerge early in viral infection^6,7^, while dysfunctional T cell emergence in chronic infections and cancers has been associated with loss of CD8^+^ T_SCM_ cells^8,9^.

CD8^+^ T_SCM_ cells are a heterogeneous population with distinct functional and developmental properties. However, persistent antigenic stimulation in chronic viral infection can disrupt memory programming. CD8^+^ T_SCM_ cells comprise two subsets: one that expresses the transcription factor TCF1 with remarkable longevity and a second progenitor type that also expresses elevated levels of the transcription factor TOX and the inhibitory receptor PD-1 (refs. ^5,10,11^). Both subsets have been identified in mice and humans, with the latter subset now recognized as precursors of exhausted CD8^+^ T cells (T_PEX_)^12,13^. The CD8^+^ T_PEX_ cell population propagates exhausted CD8^+^ T cells (T_EX_) that express high levels of multiple inhibitory immune checkpoint receptors such as PD-1, LAG3, 2B4 and TIM3. Furthermore, CD8^+^ T_EX_ cells gradually lose effector functions, including interferon-γ (IFNγ) secretion^4^. The transcription factors TCF1, ID3, BACH2 and BATF have all been shown to regulate the generation of CD8^+^ T_SCM_ cells^4,14^. However, the determinants that maintain the essential self-renewal potential of CD8^+^ T_SCM_ cells to form long-lived populations are not fully resolved.

Although readily cleared acute viral infections give rise to effector (T_EFF_) and T_M_ cells, including T_CM_ and T_SCM_ cells, persistent infections such as hepatitis virus, human immunodeficiency virus in humans and certain strains of lymphocytic choriomeningitis virus (LCMV) in mice also give rise to CD8^+^ T_SCM_ cells, even though CD8^+^ T_PEX_ cell populations contribute significantly to the propagation of CD8^+^ T_EX_ cells in these chronic infections^10,15^. However, in latent herpesvirus infections, pathogen-specific T cell populations remain functional. For instance, cytomegalovirus infection can generate significantly expanded long-term stable CD8^+^ T cell populations in a phenomenon known as memory inflation^16^. The factors sustaining inflationary CD8^+^ T cells remain unclear.

The transcriptional repressor GFI1 is highly expressed in thymocytes following T cell receptor (TCR) activation, and loss of GFI1 impairs early T cell development in the thymus^17^. In mature T cells, GFI1 deficiency differentially affects CD4^+^ and CD8^+^ T cell subsets, with GFI1-deficient CD4^+^ T cells exhibiting aberrant proliferation, while *Gfi1*^–/–^ CD8^+^ T cells are not impaired^18^. Antiviral CD8^+^ T cells in *Gfi1*^–/–^ mice show elevated IL-7R expression^19^, suggesting that a lack of GFI1 may enhance IL-7-dependent proliferation. However, the exact role of GFI1 in maintaining T_M_ cells following acute and chronic viral infection is not understood.

Here, we examined the role of GFI1 in CD8^+^ T_M_ cell development after acute and chronic viral infection. GFI1 was differentially expressed across T_M_ and T_EFF_ cells resolving GFI1^hi^ and GFI1^lo^ populations. GFI1^hi^ cells were transcriptionally distinct from GFI1^lo^ cells, expressing a memory phenotype and a superior ability to generate long-lived T_M_ cells. Ablation of GFI1 early in chronic infection showed that antiviral CD8^+^ T cells could still develop, whereas CD8^+^ T_SCM_ cells were lost. Altogether, our data identify that GFI1 is a key regulator of the fitness of CD8^+^ T_SCM_ cells, which maintain memory compartments in chronic infection and enable replenishment of effector populations.

## Results

### GFI1 is selectively expressed by T_M_ cells

**Fig. 1:**
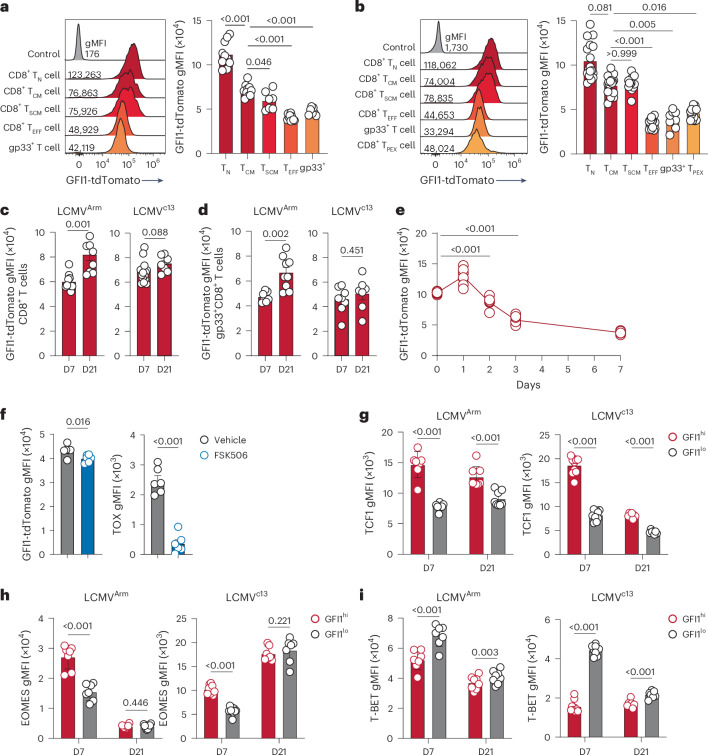
GFI1 is differentially expressed in CD8^+^ T cell effector subsets during chronic infection. **a**, Representative histogram (left) and quantification (right) of GFI1-tdTomato expression in splenic CD11a^–^CD44^–^CD8^+^ T_N_ cells from C57BL/6 mice (control) and CD8^+^ T_N_, CD11a^+^CD44^+^CD62L^+^CD27^+^CD8^+^ T_CM_ cells, CD11a^+^CD44^+^TCF1^+^CX3CR1^–^CD8^+^ T_SCM_ cells, TCF1^-^CX3CR1^+^CD8^+^ T_EFF_ cells and gp33^+^CD8^+^ T cells from the spleen of *Gfi1*^*tdTomato*/+^ mice at day 7 (D7) post infection with LCMV^Arm^. Data are pooled from two experiments (*n* = 7 mice). gMFI, geometric mean fluorescence intensity. **b**, Representative histogram (left) and quantification (right) of GFI1-tdTomato expression in splenic CD8^+^ T_N_ cells from C57BL/6 mice (control) and CD8^+^ T_N_, CD8^+^ T_CM_, CD8^+^ T_SCM_, CD8^+^ T_EFF_, CD8^+^gp33^+^ and TCF1^+^TOX^+^CD8^+^ T_PEX_ cells from the spleen of LCMV^c13^-infected *Gfi1*^*tdTomato*/+^ mice at D7. Pooled from four experiments (*n* = 13 mice). **c**,**d**, GFI1-tdTomato in total CD8^+^ T cells (**c**) and gp33^+^CD8^+^ T cells (**d**) from the spleen of LCMV^Arm^ and LCMV^c13^-infected *Gfi1*^*tdTomato*/+^ mice at D7 and D21. Pooled from two experiments (*n* = 8 or 17 mice per time point). **e**,**f**, Expression of GFI1-tdTomato in splenic CD8^+^ P14 T cells at D0–D7 post LCMV^c13^ infection (*n* = 8 mice per time point) (**e**) and GFI1-tdTomato and TOX in splenic CD8^+^ P14 T cells at D7 post LCMV^c13^ infection (**f**) in C57BL/6 mice transferred with congenically labeled *Gfi1*^*tdTomato*/+^ CD8^+^ P14 T_N_ cells, infected with LCMV^c13^ 1 day later (**e**,**f**) and treated with FK506 or vehicle daily from D4–D6 post infection (**f**). Pooled from two experiments (*n* = 6 mice). **g**–**i**, TCF1 (**g**), EOMES (**h**) and T-BET (**i**) expression in activated CD11a^+^CD44^+^GFI1^hi^ and CD11a^+^CD44^+^GFI1^lo^ CD8^+^ T cells from LCMV^Arm^-infected and LCMV^c13^-infected *Gfi1*^*tdTomato*/+^ mice at D7 and D21 post infection. Data pooled from two experiments (*n* = 7 or 8 mice). Individual values and means are shown; error bars, s.e.m. Error bars not shown in **e**. Statistical significance using a one-way ANOVA and Dunnett’s post hoc test for **a**, **b** and **e;** two-tailed Student’s *t*-test for **c**, **d** and **f**; Wilcoxon signed-rank two-tailed paired *t*-test for **g**–**i**.

**Extended Data Fig. 1:**
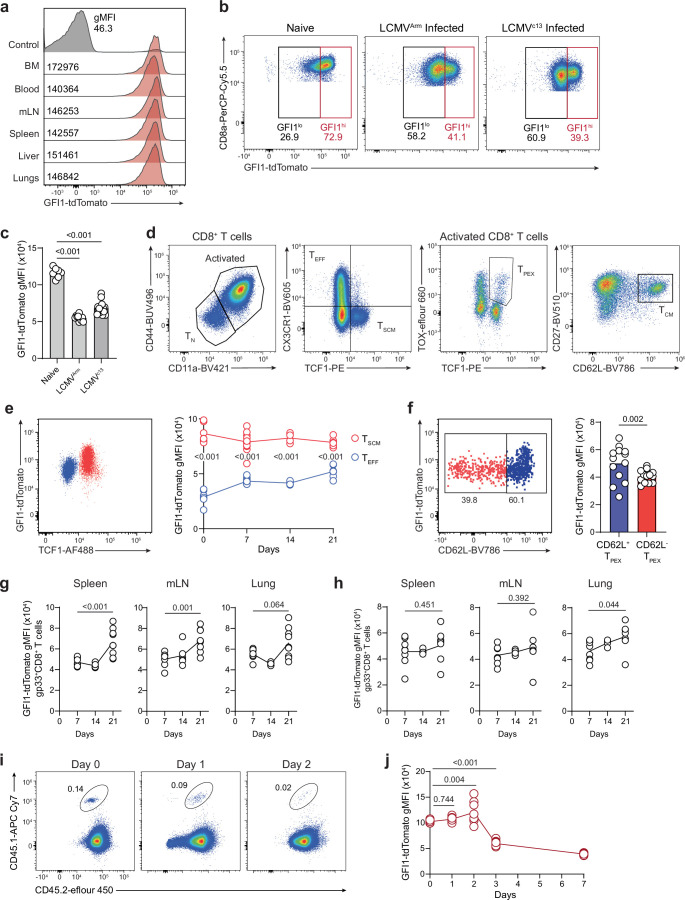
GFI1 expression in antiviral CD8^+^ T cell following virus infection. **a**, Expression of GFI1-tdTomato in naïve CD8^+^ T cells (live CD45^+^CD3^+^TCRb^+^CD8a^+^) isolated from different tissues of naïve *Gfi1*^*tdTomato*^ mice. Control CD8^+^ T cells were isolated from the spleen of a naïve C57BL/6 mouse. Histograms show geometric mean florescence intensity (gMFI) of GFI1-tdTomato expression. Data representative of three independent experiments except for the bone marrow (BM) data which is from a single experiment. **b**, Flow cytometric dot plots showing frequency of GFI1^hi^ and GFI1^lo^ cell among total CD8^+^ T cells (live CD3^+^TCRb^+^CD8a^+^) isolated from spleen of naïve, LCMV^Arm^- or LCMV^c13^-infected *Gfi1*^*tdtomato*^ mice at D7 after infection. **c**, GFI1-tdTomato expression in total CD8^+^ T cells isolated from spleen of naïve, LCMV^Arm^- or LCMV^c13^-infected *Gfi1*^*tdtomato*^ mice at D7 after infection. Data are pooled from two experiments (*n* = 7, 10 or 15 mice/condition). *p* values were calculated using a two-tailed unpaired Student’s *t* test. **d**, Gating strategy for naïve (T_N_, CD11a^–^CD44^–^) and activated (CD11a^+^CD44^+^) CD8^+^ T cells among total CD8^+^ T cells (live CD45^+^CD3^+^CD8a^+^). Activated CD8^+^ T cells were further separated into T_CM_ (CD27^+^CD62L^+^), T_SCM_ (TCF1^+^CX3CR1^-^), T_EFF_ (TCF1^-^CX3CR1^+^) and T_PEX_ (TCF1^+^TOX^+^) populations. **e**, Flow cytometric dot plots showing T_SCM_ (red dots) and T_EFF_ (blue dots) cells isolated from spleen at D7 after LCMV^c13^ infection (left panel). GFI1-tdTomato expression in T_SCM_ and T_EFF_ CD8^+^ T cells isolated from spleen of LCMV^c13^ infected mice (right panel). Data pooled from two experiments (D0, D7 and D21, *n* = 6, 7 or 13 mice/timepoint), or a single experiment (D14, *n* = 4 mice) and show individual responses. *p* values were determined using the Wilcoxon signed-rank paired *t* test. **f**, Frequency of CD62L^+^ T_PEX_ (blue dots) and CD62L^–^ T_PEX_ (red dots) among total CD8^+^ T_PEX_ T cells isolated from spleen at D7 after LCMV^c13^ infection (left panel). GFI1-tdTomato expression in CD62L^+^ and CD62L^–^ T_PEX_ CD8^+^ T cells isolated from spleen at D7 (right panel). *p* values were calculated using a Wilcoxon signed-rank paired *t* test. Data are pooled from two independent experiments (*n* = 13 mice). **g, h**, GFI1-tdTomato expression in gp33^+^CD8^+^ T cells in spleen, mLN and lungs following LCMV^Arm^ (**g**) or LCMV^c13^ (**h**) infection. Data pooled from two experiments (D7 and D21, *n* = 6, 7 or 8 mice/timepoint), or a single experiment (D14 for panel **h**, *n* = 4 mice) and show individual responses. *p* values were calculated using a two-tailed unpaired Student’s *t* test. **i,j**, Naïve *Gfi1*^*tdTomato*^ P14 T cells (CD45.1^+^CD45.2^+^, 1×10^5^ cells) were transferred to congenic recipients (CD45.2^+^) and the next day recipients were infected with LCMV^c13^. **i**, Flow cytometric plots showing P14 T cells frequency among total CD8^+^ T cells following LCMV^c13^ infection. **j**, Expression of GFI1-tdTomato by P14 T cells isolated from mLN following infection. *p* values were calculated using one-way ANOVA and Dunnett’s post-test. Data pooled from two independent experiments (*n* = 8 mice/ timepoint).

To understand how GFI1 is regulated in CD8^+^ T cells, *Gfi1*^*tdTomato*/+^ reporter mice, which express tdTomato under the *Gfi1* promotor in all cells^20–22^, were infected with LCMV that induces either acute infection (Armstrong strain, LCMV^Arm^) or chronic infection (clone 13 strain, LCMV^c13^). In naive *Gfi1*^*tdTomato*/+^ mice, GFI1-tdTomato was uniformly highly expressed in CD8^+^ T cells (Extended Data Fig. 1a). Following infection, CD8^+^ T cells showed a significant downregulation of GFI1-tdTomato expression (Extended Data Fig. 1b,c). Further analyses revealed a graded expression of GFI1-tdTomato whereby CD8^+^ T_CM_ and CD8^+^ T_SCM_ cells exhibited high expression of GFI1-tdTomato (Fig. 1a,b and Extended Data Fig. 1d). TCF1^−^CX3CR1^+^ T_EFF_ cells showed the lowest GFI1-tdTomato expression (Fig. 1a,b and Extended Data Fig. 1e), whereas high expression was maintained in CD8^+^ T_SCM_ cells (Extended Data Fig. 1e). TCF1^+^TOX^+^ CD8^+^ T_PEX_ cells that maintain stem-like proliferation following LCMV^c13^ infection^4^ showed significantly lower expression of GFI1-tdTomato than CD8^+^ T_CM_ and CD8^+^ T_SCM_ cells (Fig. 1b). Further resolving the CD8^+^ T_PEX_ cell population revealed that CD62L^+^CD8^+^ T_PEX_ cells, which were reported to exhibit superior proliferative capacity^23^, had higher GFI1-tdTomato expression than CD62L^−^CD8^+^ T_PEX_ cells (Extended Data Fig. 1f). Following the initial downregulation of GFI1-tdTomato expression after LCMV infection, GFI1-tdTomato subsequently increased between day 7 and day 21 for LCMV^Arm^ infection but remained low following LCMV^c13^ infection (Fig. 1c). This pattern persisted in LCMV-specific gp33^+^CD8^+^ T cells during late LCMV^c13^ infection (Fig. 1d and Extended Data Fig. 1g,h). Tracking the temporal dynamics of GFI1 expression early in infection using *Gfi1*^*tdTomato*/+^ P14 CD8^+^ T cells showed that GFI1-tdTomato expression was significantly downregulated on day 2 and day 3 in P14 T cells isolated from spleen and mesenteric lymph nodes (mLN), respectively (Fig. 1e and Extended Data Fig. 1i,j). FK506, a potent inhibitor of calcineurin that blocks TCR-dependent signaling, showed that although TOX expression was impaired, GFI1-tdTomato was not affected (Fig. 1f). Thus, calcineurin signaling was not essential to GFI1 regulation, and infection-induced inflammation was sufficient for GFI1 downregulation. Together, these data show that GFI1 is rapidly downregulated in activated CD8^+^ T cells after infection and is selectively maintained in T_M_ cell subsets.

Given the emergence of distinct high and low GFI1-tdTomato-expressing populations among activated CD11a^+^CD44^+^ CD8^+^ T cells, the GFI1^hi^ and GFI1^lo^ CD8^+^ T cells (Extended Data Fig. 1b) were further analyzed to determine the expression of other transcription factors. GFI1^hi^CD8^+^ T cells showed increased expression of TCF1 and EOMES compared to GFI1^lo^CD8^+^ T cells at day 7 post LCMV^Arm^ or LCMV^c13^ infection (Fig. 1g,h). TCF1 remained high in GFI1^hi^CD8^+^ T cells at day 21, while EOMES expression was similar in GFI1^hi^ and GFI1^lo^ CD8^+^ T cells at day 21 (Fig. 1h). T-BET expression was low in GFI1^hi^CD8^+^ T cells at day 7 and day 21 after LCMV^Arm^ and LCMV^c13^ infection (Fig. 1i). Therefore, GFI1 was differentially regulated among T_M_ and T_EFF_ cells and was associated with memory CD8^+^ T cell formation.

### GFI1 identifies transcriptionally distinct CD8^+^ T cells

**Fig. 2:**
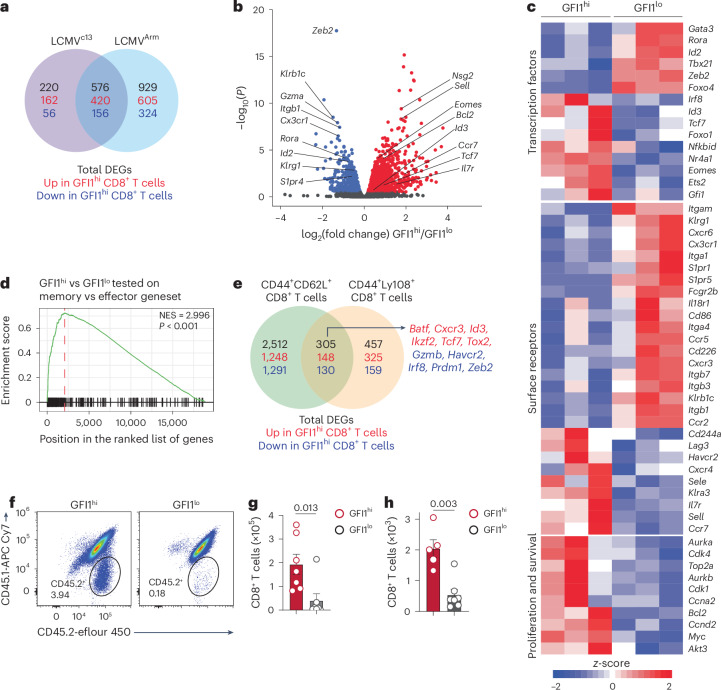
GFI1-expressing CD8^+^ T cells exhibit a T_M_ cell molecular program. **a**, Shared and unique DEGs (*P* < 0.05) in activated CD11a^+^CD44^+^GFI1^hi^ CD8^+^ T cells isolated from the spleen of *Gfi1*^*tdTomato*/+^ mice at D7 post infection with LCMV^c13^ or LCMV^Arm^ and analyzed by RNA-seq. **b**, Volcano plot of DEGs in GFI1^hi^ CD8^+^ T cells from LCMV^c13^-infected mice as in **a**. Blue and red indicate genes upregulated in activated GFI1^lo^ and GFI1^hi^ CD8^+^ T cells, respectively. Gray dots indicate *P* > 0.05. **c**, DEseq2-normalized expression of selected genes in GFI1^hi^ and GFI1^lo^ CD8^+^ T cells isolated from LCMV^c13^-infected mice as in **a**. **d**, Gene set enrichment analysis of GFI1^hi^ and GFI1^lo^ CD8^+^ T cells isolated from LCMV^c13^-infected mice as in **a**, using a memory CD8^+^ T cells gene signature^26^. NES, normalized enrichment score. **e**, Shared and unique DEGs (*P* < 0.05) in CD44^+^CD62L^+^GFI1^hi^ and CD44^+^Ly108^+^GFI1^hi^ CD8^+^ T_M_ cells isolated from the spleen of *Gfi1*^*tdTomato*/+^ mice at D7 post infection with LCMV^c13^ and analyzed by RNA-seq. **f**,**g**, Representative flow cytometry plots (**f**) and quantification (**g**) of spleen donor CD45.2^+^CD8^+^ T cells at D7 post LCMV^Arm^ infection in congenic C57BL/6 mice transferred intravenously (i.v.) with splenic CD8^+^ T cells isolated at D21 post LCMV^Arm^ infection from CD45.2^+^ *Gfi1*^*tdTomato*/+^ mice and infected with LCMV^Arm^ 24 h later. Data pooled from two experiments (*n* = 7 mice per group). **h**, Total donor CD45.2^+^CD8^+^ T cells at D14 post LCMV^c13^ infection in the spleen of congenic C57BL/6 recipients infected with LCMV^c13^ 7 days before transfer of infection-matched (D7) CD8^+^ T cells isolated from LCMV^c13^-infected CD45.2^+^ *Gfi1*^*tdTomato*/+^ mice. Data pooled from two experiments (*n* = 5 or 7 mice per group). *P* values were calculated using a Wald’s test (**b**, **d** and **e**) or a two-tailed Student’s *t*-test (**g** and **h**). Data in **g** and **h** show means; error bars, s.e.m.

**Extended Data Fig. 2:**
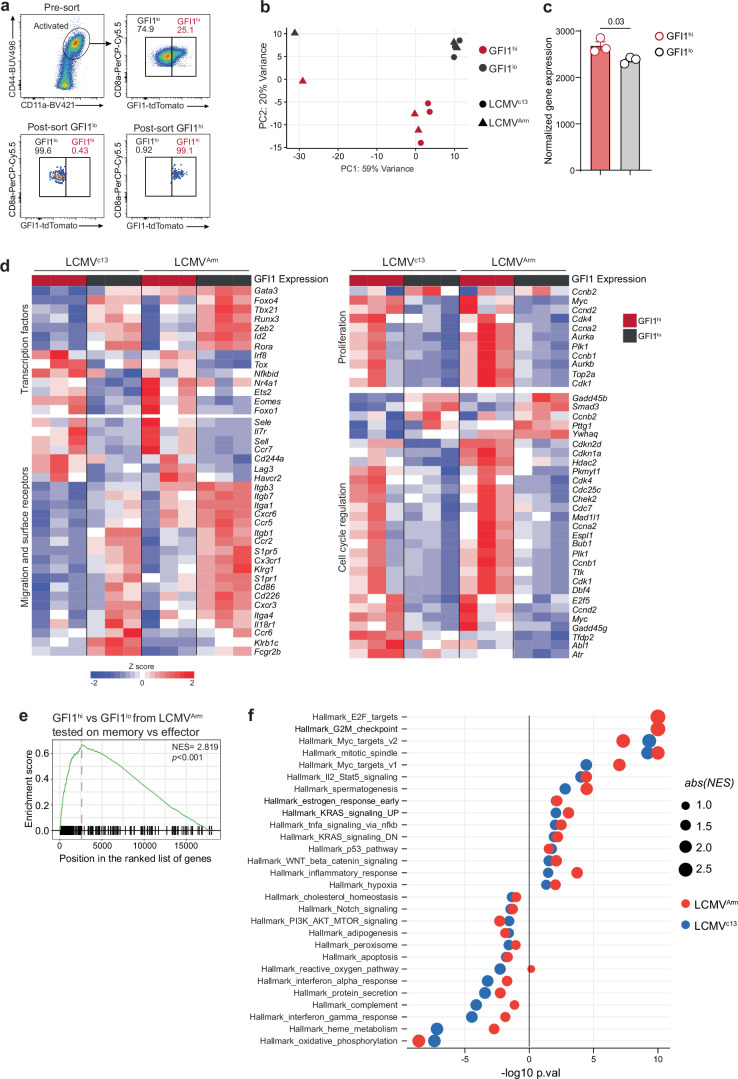
Transcriptional profile of antiviral GFI1^high^ and GFI1^low^ CD8^+^ T cells following chronic or acute LCMV infection. *Gfi1*^*tdTomato/+*^ mice were infected with LCMV^c13^ or LCMV^Arm^ virus *via* tail vein or intraperitoneal injection, respectively. Activated (CD11a^+^CD44^+^) GFI1^hi^ and GFI1^lo^ CD8^+^ T cells were flow cytometrically sorted at D7 after infection and RNA-seq was performed. **a**, Gating strategy used to sort GFI1^hi^ and GFI1^lo^ CD8^+^ T cells from spleen of infected *Gfi1*^*tdTomato/+*^ mice at D7. **b**, Principal component analyses (PCA) plot showing clustering of CD8^+^ T cells. Replicates of the same group are indicated by the same colour and shape. **c**, DEseq2 normalized gene expression for *Gfi1* in GFI1^hi^ and GFI1^lo^ CD8^+^ T cells following LCMV^c13^ infection. *p* value was calculated using two-tailed Student’s *t* test. **d**, DEseq2 normalized expression of selected genes in CD8^+^ T cells isolated from LCMV^c13^ or LCMV^Arm^ infected *Gfi1*^*tdTomato*^ mice. **e**, GSEA analysis of CD8^+^ T cells isolated from LCMV^Arm^-infected mice tested on memory CD8^+^ T cells gene signature^26^. *p* value was calculated using Wald’s test. **f**, GSEA Hallmark pathway analysis. Dot plots show the changes in GSEA hallmark pathways, where size of dot represents absolute pathway enrichment in GFI1^hi^ *vs* GFI1^lo^ comparison. *p* values were calculated using Wald’s test.

**Extended Data Fig. 3:**
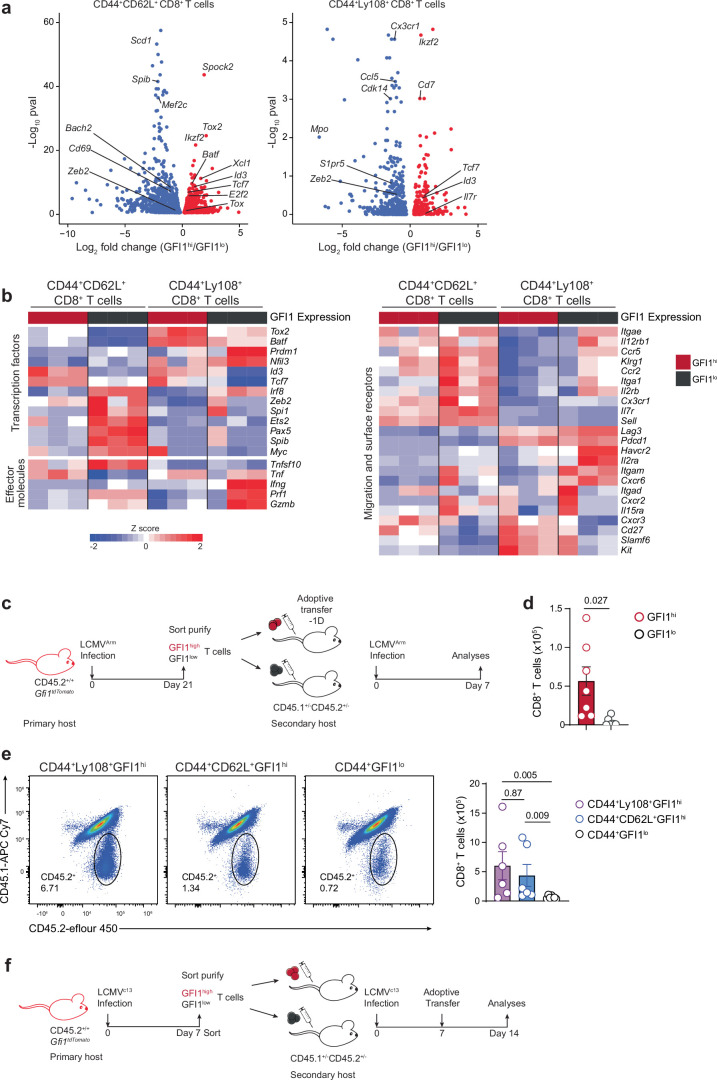
Transcriptional profile of GFI1^high^ central memory and stem cell memory CD8^+^ T cells. **a,b**, *Gfi1*^*tdTomato/+*^ mice were infected with LCMV^c13^ via tail vein injection. GFI1^hi^ and GFI1^lo^ CD8^+^ T cells from CD44^+^CD62L^+^ (T_CM_) and CD44^+^Ly108^+^ (T_SCM_ precursor) CD8^+^ T cells were flow cytometrically sorted from spleen and mLN at D7 after infection and RNA-seq was performed. *p* values were calculated using Wald’s test. **a**, Volcano plot showing DEGs (*p* < 0.05) in CD44^+^CD62L^+^ and CD44^+^Ly108^+^ GFI1^hi^ CD8^+^ T cells isolated from LCMV^c13^-infected mice. Blue and red dots show genes upregulated in GFI1^lo^ and GFI1^hi^ CD8^+^ T cells, respectively. **b**, DEseq2 normalized expression of selected genes in CD44^+^CD62L^+^ and CD44^+^Ly108^+^ CD8^+^ T cells isolated from LCMV^c13^-infected *Gfi1*^*tdTomato*^ mice. **c**, Schematic of *Gfi1*^*tdTomato/+*^ CD8^+^ T cell isolation and transfer (1×10^5^ cells/recipient) to congenic mice followed by challenge with LCMV^Arm^ infection. **d**, Total donor cells in mLN of recipients at D7 after LCMV^Arm^ infection. Data pooled from two experiments (*n* = 7 mice/group). *p* value calculated using two-tailed Student’s *t* test. **e**, CD44^+^Ly108^+^GFI1^hi^, CD44^+^CD62L^+^GFI1^hi^ or CD44^+^GFI1^lo^ CD8^+^ T cells were flow cytometrically isolated from spleen of *Gfi1*^*tdTomato*^ mice (CD45.2^+^) at D21 after LCMV^Arm^ infection. Each population was adoptively transferred (5×10^4^ cells) into congenic recipient mice (CD45.1^+^CD45.2^+^ or CD45.1^+^), then infected with LCMV^Arm^. Flow cytometric dot plots show frequency of donor cells (CD45.2^+^) among total CD8^+^ T cells isolated from spleen at D7 after LCMV^Arm^ infection (left plots). Total number of donor cells in spleen of recipients at D7 after LCMV^Arm^ infection. Data are pooled from two independent experiments (*n* = 6 or 7 mice/group). *p* values were calculated using Kruskal-Wallis test followed by Benjamini–Hochberg FDR test for individual comparison. **f**, Schematic of *Gfi1*^*tdTomato/+*^ CD8^+^ T cell isolation and transfer (1×10^5^ cells/recipient) to infection matched mice.

RNA sequencing (RNA-seq) of GFI1^hi^ and GFI1^lo^ CD8^+^ T cells isolated from LCMV^Arm^-infected or LCMV^c13^-infected mice (Extended Data Fig. 2a) showed that GFI1^hi^CD8^+^ T cells responding to both infections exhibited a similar transcriptional profile, which was distinct from that of GFI1^lo^CD8^+^ T cells (Extended Data Fig. 2b). We identified 929 and 220 unique differentially expressed genes (DEGs) in GFI1^hi^CD8^+^ T cells isolated from LCMV^Arm^-infected and LCMV^c13^-infected mice, respectively (Fig. 2a and Supplementary Tables 1 and 2). GFI1^hi^CD8^+^ T cells showed upregulation of transcription factors associated with T cell memory^4,24^, including *Eomes*, *Id3* and *Tcf7*, whereas transcription factors linked with T_EFF_ cell programs such as *Id2*, *Tbx21* and *Zeb2* were downregulated^24,25^ (Fig. 2b,c). GFI1^hi^CD8^+^ T cells showed higher *Gfi1* expression than GFI1^lo^ CD8^+^ T cells (Fig. 2c and Extended Data Fig. 2c), suggesting that the reporter levels reflect GFI1 gene expression. GFI1^hi^CD8^+^ T cells showed upregulation of genes associated with cell proliferation and cell cycle, including *Cdk1*, *Top2a* and *Myc* (Fig. 2c and Extended Data Fig. 2d) and significant enrichment of memory signature genes, such as *Tcf7*, *Eomes* and *Il7r* (Fig. 2d and Extended Data Fig. 2e), suggesting that GFI1^hi^CD8^+^ T cells had enhanced proliferative potential. This premise was further supported by upregulation of *E2f* target genes and the G2M checkpoint pathway in GFI1^hi^CD8^+^ T cells (Extended Data Fig. 2f). To understand whether the memory signature^26^ observed in the transcriptome of GFI1^hi^CD8^+^ T cells was solely caused by enrichment of CD8^+^ T_CM_ and CD8^+^ T_SCM_ cell subsets or whether GFI1-tdTomato expression regulated memory gene expression within CD8^+^ T_CM_ and CD8^+^ T_SCM_ cell populations, the transcriptional profile of GFI1^hi^ and GFI1^lo^ subsets from CD44^+^CD62L^+^ and CD44^+^Ly108^+^ (Ly108 was used as a surrogate marker for TCF1^+^ memory cells^27^) CD8^+^ T cell populations were analyzed. We found that GFI1^hi^CD8^+^ T cell fractions within CD8^+^ T_CM_ and CD8^+^ T_SCM_ cells expressed higher levels of *Id3*, *Ikzf2* and *Tcf7*, whereas *Gzmb*, *Havcr2* and *Zeb2* showed lower expression (Fig. 2e, Extended Data Fig. 3a,b and Supplementary Tables 3 and 4). These data strongly suggest that high expression of GFI1 identifies key features of memory programmed CD8^+^ T cells, indicating a superior capacity to respond to infection.

To determine whether GFI1^hi^ and GFI1^lo^ CD8^+^ T cells differed in their capacity to respond to a secondary infection, GFI1^hi^ and GFI1^lo^ CD8^+^ T cells were isolated from the spleen of LCMV^Arm^-infected wild-type (WT) mice on day 21 after infection and adoptively transferred into secondary recipients that were challenged with LCMV^Arm^ the next day (Extended Data Fig. 3c). In this acute challenge model, GFI1^hi^ CD8^+^ T cells expanded approximately fourfold more than GFI1^lo^ CD8^+^ T cells by day 7 post LCMV^Arm^ infection (Fig. 2f,g and Extended Data Fig. 3d), demonstrating the enhanced proliferative potential of GFI1^hi^ CD8^+^ T cells in response to a secondary infection. Next, we adoptively transferred CD44^+^CD62L^+^GFI1^hi^, CD44^+^Ly108^+^GFI1^hi^ or CD44^+^GFI1^lo^ CD8^+^ T cells isolated from spleen and mLN of LCMV^Arm^-infected mice at day 21 after infection into congenically marked secondary recipient mice that were subsequently infected with LCMV^Arm^ the next day. This procedure showed that both CD44^+^CD62L^+^GFI1^hi^ and CD44^+^Ly108^+^GFI1^hi^ CD8^+^ T cell populations exhibited superior expansion compared with that of CD44^+^GFI1^lo^CD8^+^ T cells (Extended Data Fig. 3e). In line with these observations, adoptive transfer of GFI1^hi^ and GFI1^lo^ CD8^+^ T cells isolated from spleen of *Gfi1*^*tdTomato*/+^ mice infected with LCMV^c13^ at day 7 after infection into infection-matched recipients (Extended Data Fig. 3f) showed that GFI1^hi^CD8^+^ T cells had more enhanced capacity to proliferate than GFI1^lo^ CD8^+^ T cells (Fig. 2h). Collectively, these data showed that GFI1^hi^ CD8^+^ T cells identified a T_M_ cell population with superior expansion and recall response capacity.

### GFI1 epigenetically regulates CD8^+^ T cell function and persistence

**Fig. 3:**
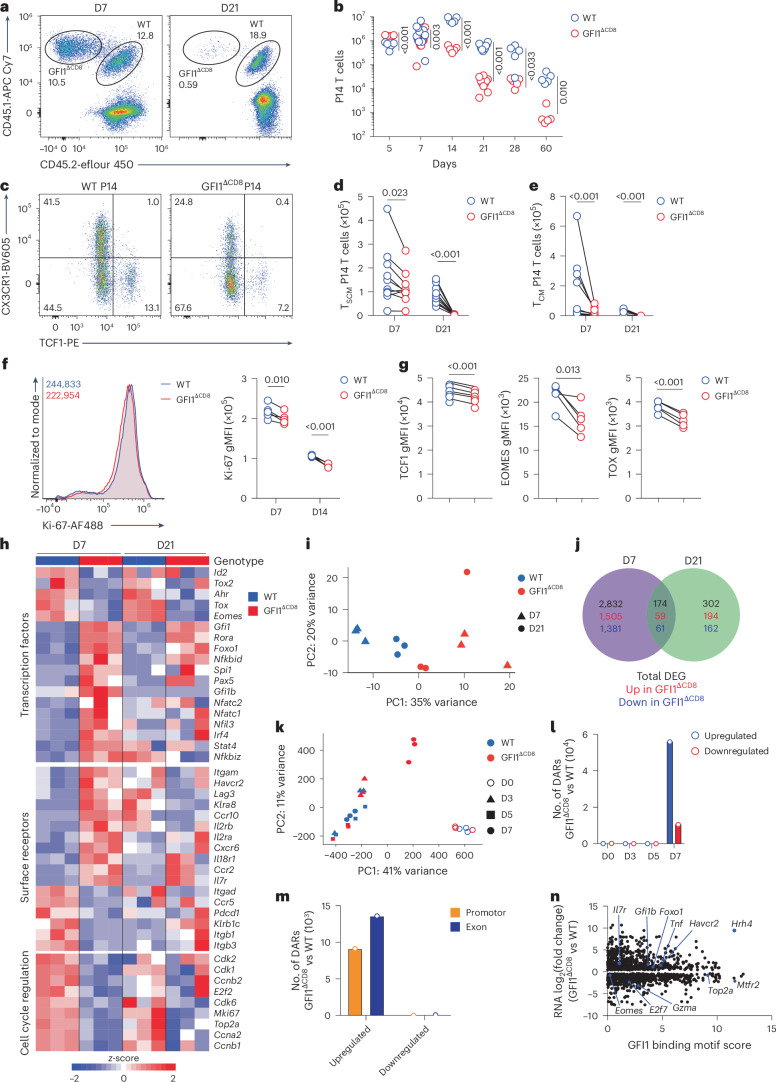
GFI1 drives epigenetic and transcriptional changes to promote antiviral CD8^+^ T cell persistence. **a**,**b**, Representative flow cytometry plots (**a**) and total number (**b**) of WT and GFI1^ΔCD8^ live CD8^+^ P14 T cells at D5–D60 post infection in the spleen of C57BL/6 mice that received a 1:1 mix of congenically labeled WT and GFI1^ΔCD8^ T_N_ cells followed by LCMV^c13^ infection 24 h later. Data are pooled from two (*n* = 10 mice for D7 and D21) or one experiment (*n* = 5 for D5, D14, D28 and D60). **c**, Representative plots showing frequency of CD8^+^ T_SCM_ cells at D7 in the spleen of LCMV^c13^-infected mice as in **a**. **d**,**e**, Total number of CD8^+^ P14 T_SCM_ (**d**) and CD8^+^ P14 T_CM_ (**e**) cells in the spleen of LCMV^c13^-infected mice as in **a**. Data are pooled from two experiments (*n* = 10 mice per time point). **f**, Representative histograms at D7 (right) and quantification (left) of Ki-67 expression in WT and GFI1^ΔCD8^ CD8^+^P14 T cells as in **a**. D7 data are representative of two experiments (*n* = 5 mice); D14 data are representative of one experiment. **g**, TCF1, EOMES and TOX expression in CD8^+^ P14 T cells at D7 in the spleen of LCMV^c13^-infected mice. Data are representative of two experiments (*n* = 6 mice). **h**, Expression of selected genes in WT and GFI1^ΔCD8^ CD8^+^ P14 T cells from the spleen of C57BL/6 mice transferred i.v. with congenically marked WT or GFI1^ΔCD8^ CD8^+^ P14 T_N_ cells, infected with LCMV^c13^ 24 h post transfer and analyzed by RNA-seq at D7 and D21 post LCMV^c13^ infection. **i**, PCA plot of WT or GFI1^ΔCD8^ CD8^+^P14 T cells at D7 and D21 post LCMV^c13^ infection based on RNA-seq as in **h**. **j**, Shared and unique DEGs at D7 and D21in GFI1^ΔCD8^ CD8^+^ T cells as in **h**. **k**, ATAC-seq PCA plot of splenic WT and GFI1^ΔCD8^ CD8^+^ T cells at D0, D3, D5 and D7 post LCMV^c13^ infection in C57BL/6 mice transferred i.v. with congenically labeled WT or GFI1^ΔCD8^ CD8^+^ P14 T cells 24 h before infection. **l**, Total number of DARs detected in GFI1^ΔCD8^ CD8^+^ P14 T cells versus WT CD8^+^ P14 T cells at D0, D3, D5 and D7 post LCMV^c13^ infection as in **k**. **m**, Number of DARs annotated in promotor or exon regions in GFI1^ΔCD8^ CD8^+^ P14 T cells compared with WT CD8^+^ P14 T cells as in **l**. **n**, Predicted GFI1 binding motif by HOMER analysis using DEGs from GFI1^ΔCD8^ CD8^+^ T cells isolated at D7 post LCMV^c13^ infection as in **h**. Statistical significance was calculated using a two-tailed paired *t*-test (**b** and **d**–**g**) or Wald’s test (**j**, **l** and **m**).

**Extended Data Fig. 4:**
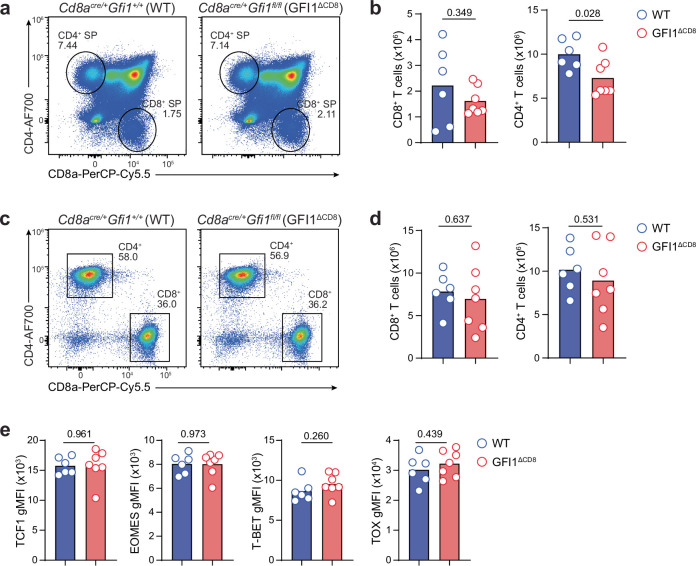
Phenotypic characterisation of naïve GFI1^ΔCD8^ CD8^+^ T cells. **a**, Frequency of CD4^+^ single positive (SP) and CD8^+^ SP cells among total live cells isolated from thymus of naïve age matched *Cd8a*^*cre/+*^*Gfi1*^*+/+*^ (WT) and *Cd8a*^*cre/+*^*Gfi1*^*fl/fl*^ (GFI1^ΔCD8^) mice. **b**, Total number of CD8^+^ (live TCRb^+^CD4^-^CD8^+^) and CD4^+^ (live TCRb^+^CD4^+^CD8^-^) T cells in thymus of naïve *Cd8a*^*cre/+*^*Gfi1*^*+/+*^ (WT) and *Cd8a*^*cre/+*^*Gfi1*^*fl/fl*^ (GFI1^ΔCD8^) mice. **c**, Frequency of CD4^+^ and CD8^+^ T cells among total T cells (live TCRb^+^CD3^+^) isolated from spleen of naïve *Cd8a*^*cre/+*^*Gfi1*^*+/+*^ (WT) and *Cd8a*^*cre/+*^*Gfi1*^*fl/fl*^ (GFI1^ΔCD8^) mice. **d**, Total number of CD8^+^ (live CD3^+^CD8^+^) and CD4^+^ (live CD3^+^CD4^+^) T cells in spleen of naïve *Cd8a*^*cre/+*^*Gfi1*^*+/+*^ (WT) and *Cd8a*^*cre/+*^*Gfi1*^*fl/fl*^ (GFI1^ΔCD8^) mice. **e**, TCF1, EOMES, T-BET and TOX expression in naïve CD8^+^ T cells isolated from spleen of naïve *Cd8a*^*cre/+*^*Gfi1*^*+/+*^ (WT) and *Cd8a*^*cre/+*^*Gfi1*^*fl/fl*^ (GFI1^ΔCD8^) mice. **b**, **d**, and **e**, data pooled from two independent experiments (*n* = 6 or 7 mice/genotype). *p* values were determined using a two-tailed Student’s *t* test.

**Extended Data Fig. 5:**
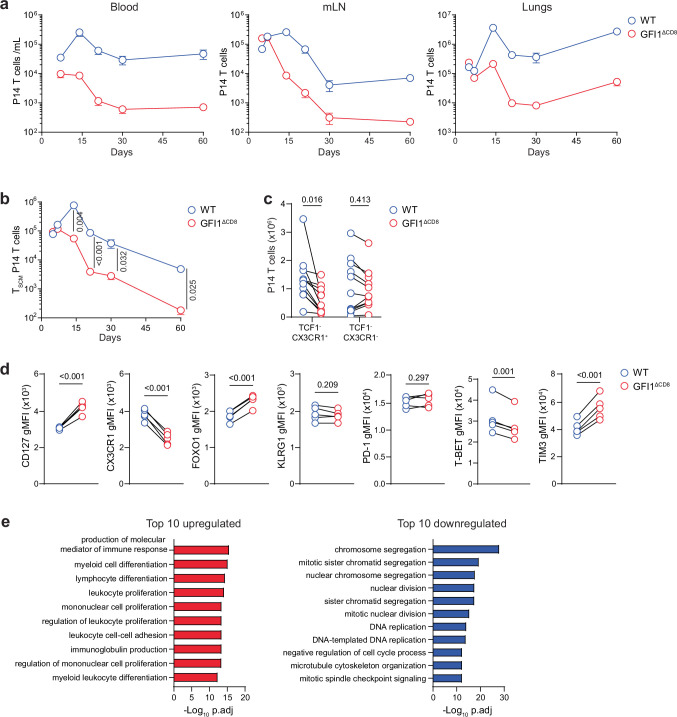
CD8^+^ T cell responses mounted by GFI1-deficient following chronic LCMV infection. **a**-**e**, Congenically-labelled naïve WT (CD45.1^+^) and GFI1^ΔCD8^ (CD45.1^+^CD45.2^+^) P14 T cells were co-transferred (5×10^3^ cells each) to C57BL/6 mice that were subsequently infected with LCMV^c13^ 24 h later. **a**, Total number of WT and GFI1^ΔCD8^ P14 T cells in peripheral blood, mLN and Lungs. **b**, Total number of T_SCM_ P14 T cells in spleen. **a**, **b**, Data pooled from two (*n* = 10 mice for D7 and D21) or one experiment (*n* = 5 for D5, D14, D28 and D60). **c**, Total number of TCF1^-^CX3CR1^+^ and TCF1^-^CX3CR1^-^ P14 T cells in spleen at D7 after infection. Data are pooled from two independent experiments (*n* = 11 mice). **d**, CD127, CX3CR1, FOXO1, KLRG1, PD-1, TIM3 and T-BET expression in WT and GFI1^ΔCD8^ P14 T cells at D7 after LCMV^c13^ infection. Data show expression from one of two independent experiments (*n* = 6 mice). **e**, Genes differentially expressed between GFI1^ΔCD8^ and WT P14 T cells were subjected to GO pathway enrichment analysis. Bar plots show top 10 significantly upregulated and downregulated pathways. *p* values calculated using Wald’s test followed by Benjamini–Hochberg FDR testing. **a**, **b**, data show mean ± s.e.m. **b**-**e** *p* values were determined with a two-tailed paired *t* test.

To study the role of GFI1, *Gfi1*^fl/fl^ mice were crossed with *CD8a*^*cre*/+^ (E8I-Cre)^28^ to create GFI1^ΔCD8^ mice, in which GFI1 was deleted in mature peripheral CD8^+^ T cells. Unlike T cells in the *Gfi1*^–/–^ strain^18^, the numbers of CD4^+^ and CD8^+^ T cells in the thymus (Extended Data Fig. 4a,b) and spleen (Extended Data Fig. 4c,d) of naive GFI1^ΔCD8^ mice were similar to WT mice. Moreover, selective ablation of GFI1 did not alter TCF1, EOMES, T-BET or TOX expression in naive CD8^+^ T cells (Extended Data Fig. 4e). When equal numbers of WT (CD45.1^+^) and GFI1^ΔCD8^ (CD45.1^+^CD45.2^+^) P14 CD8^+^ T cells were co-transferred into congenic recipients, both WT and GFI1^ΔCD8^ P14 CD8^+^ T cells expanded equivalently by day 5 after LCMV^c13^ infection (Extended Data Fig. 5a). However, the frequency and number of GFI1^ΔCD8^ CD8^+^ T cells declined from day 7 and were largely lost by day 21 after LCMV^c13^ infection in spleen, blood, mLN and lungs (Fig. 3a,b and Extended Data Fig. 5a). This decline mirrored a reduction in P14 CD8^+^ T_SCM_ cells (Fig. 3c,d and Extended Data Fig. 5b) and P14 CD8^+^ T_CM_ cells (Fig. 3e). CD8^+^ T_EFF_ cells were also decreased at day 7 (Fig. 3c and Extended Data Fig. 5c). GFI1^ΔCD8^ P14 CD8^+^ T cells showed lower expression of Ki-67, TCF1, EOMES, TOX and CX3CR1 (Fig. 3f,g and Extended Data Fig. 5d) and increased expression of FOXO1, CD127 and TIM3 (Extended Data Fig. 5d) compared to WT P14 CD8^+^ T cells. These results demonstrate that GFI1 is needed for antigen-specific memory CD8^+^ T cell persistence in chronic infection.

**Extended Data Fig. 6:**
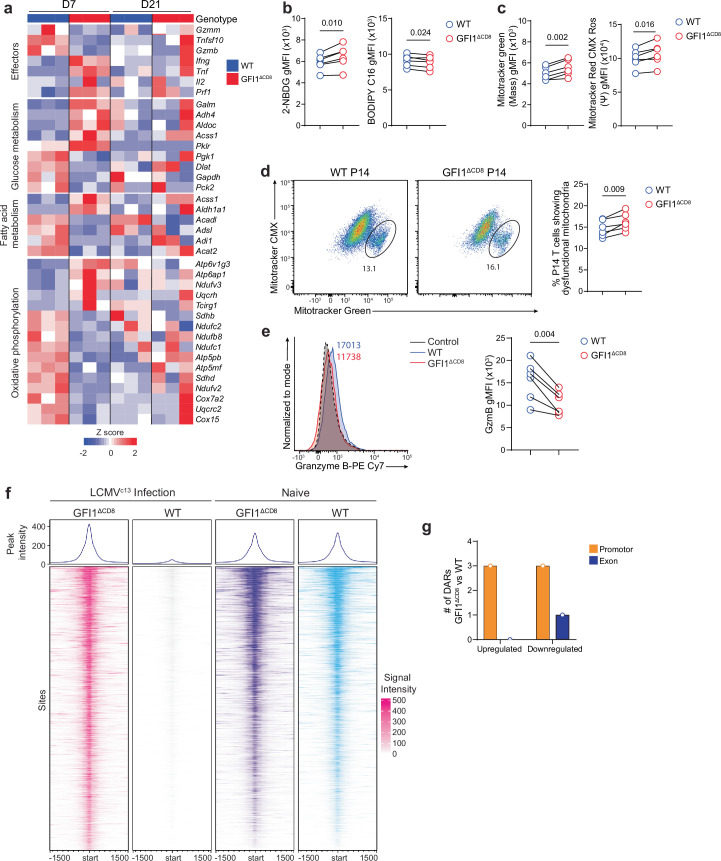
Characterization of transcriptional and epigenetic profile of GFI1-deficient CD8^+^ T cells following chronic LCMV infection. **a**, Naïve congenically labelled WT or GFI1^ΔCD8^ P14 CD8^+^ T cells (CD45.1^+^) were adoptively transferred into C57BL/6 mice that were subsequently infected with LCMV^c13^. P14 T cells were isolated from spleen at D7 after infection and analysed using RNA-seq. DEseq2 normalized expression of selected genes is shown. **b**-**e**, Congenically-labelled naïve WT (CD45.1^+^) and GFI1^ΔCD8^ (CD45.1^+^CD45.2^+^) P14 T cells were mixed 1:1 and co-transferred to C57BL/6 mice that were subsequently infected with LCMV^c13^ 24 h later. Splenic P14 T cells were analysed at D7 after infection. **b**, Uptake of fluorescent glucose analog 2-NBDG and fluorescent fatty acid (BODIPY C16) in P14 T cells isolated from spleen at D7. **c**, Mitochondrial mass and membrane potential determined by Mitotracker Green and Mitotracker Red (CMX ROS) labeling, respectively, in P14 T cells isolated at D7. **d**, Plots showing frequency of P14 cells with dysfunctional mitochondria (left panels). Data showing frequency of P14 cells with dysfunctional mitochondria (right panel). **e**, Histograms showing granzyme B expression in WT (blue line) and GFI1^ΔCD8^ (red line) P14 CD8^+^ T cells following gp33 peptide stimulation. Control (black dotted line) shows unstimulated WT P14 CD8^+^ T cells (left panel). Data show granzyme B expression in P14 T cells at D7. **b**-**e** Data are representative of two experiments (*n* = 6 mice). *p* values calculated using a two-tailed paired *t* test. **f,g**, Naïve congenically-labelled WT or GFI1^ΔCD8^ P14 CD8^+^ T cells (CD45.1^+^) were adoptively transferred into C57BL/6 mice that were infected with LCMV^c13^ 24 h later. Nuclei from activated splenic P14 T cells (D7 after infection) and naïve P14 T cells were isolated to perform bulk ATAC-seq. **f**, Chromatin accessibility read density enrichment around peak centres for P14 T cells. Upper panels show the average profile around detected peak centres. Lower panels show read intensity heatmaps ranked by total read intensity. Reads are centred on the middle of the accessible peak ±1.5 Kbp. Data pooled from three samples to generate read density map. **g**, Number of differentially accessible regions detected in naïve GFI1^ΔCD8^ P14 CD8^+^ T cells and annotated in and around promotor or exon regions.

To identify the transcriptional program regulated by GFI1 following CD8^+^ T cell activation, WT and GFI1^ΔCD8^ P14 CD8^+^ T cells were analyzed by RNA-seq on day 7 and day 21 after LCMV^c13^ infection. Loss of GFI1 impaired expression of proliferation and cell cycle genes such as *Cdk1*, *Cdk2*, *E2f2*, *Mki67* and *Top2a* (Fig. 3h) and altered transcription of memory genes including *Eomes, Id2* and *Tox* (Fig. 3h,i and Supplementary Table 5). RNA-seq identified 2,832 unique DEGs in GFI1^ΔCD8^ P14 CD8^+^ T cells at day 7 and 302 DEGs at day 21 (Fig. 3j). Pathway analyses showed downregulation of DNA replication and cell division pathways (Extended Data Fig. 5e), indicating that GFI1 had a key role in proliferation. GFI1 loss also disrupted metabolic gene expression associated with glucose and lipid uptake such as *Acss1*, *Pgk1* and *Uqcrh* (Extended Data Fig. 6a,b), increased mitochondrial dysfunction (Extended Data Fig. 6c,d) and reduced granzyme B production (Extended Data Fig. 6e).

GFI1 modifies chromatin to repress transcription^29^. Assay for transposase-accessible chromatin using sequencing (ATAC-seq) showed that WT and GFI1^ΔCD8^ P14 CD8^+^ T_N_ cells had similar epigenetic profiles (Fig. 3k,l and Extended Data Fig. 6f). By contrast, CD8^+^ T cell activation following LCMV^c13^ infection resulted in a significant change in the epigenetic profile of LCMV-activated CD8^+^ T cells compared with CD8^+^ T_N_ cells (Fig. 3k,l). Both WT and GFI1^ΔCD8^ CD8^+^ T cells showed a similar epigenetic profile on day 3 and day 5 after infection, whereas at day 7, the profile of GFI1^ΔCD8^ CD8^+^ T cells was substantially different from WT CD8^+^ T cells (Fig. 3k,l and Supplementary Table 6). Differentially accessible chromatin regions (DARs) were upregulated in GFI1^ΔCD8^ CD8^+^ T cells following activation but not in CD8^+^ T_N_ cells (Fig. 3l,m and Extended Data Fig. 6g). The HOMER motif discovery analysis predicted that the GFI1 binding motif in the promotor region of various targets is important for CD8^+^ T cell proliferation and differentiation, such as *E2f7*, *Eomes*, *Foxo1*, *Havcr2* and *Il7r* (Fig. 3n and Supplementary Table 7). Altogether, these data show that GFI1-mediated epigenetic silencing regulates the CD8^+^ T cell transcriptional program to promote their persistence during chronic viral infection.

### Loss of GFI1 abrogates inflationary CD8^+^ T cell responses

**Fig. 4:**
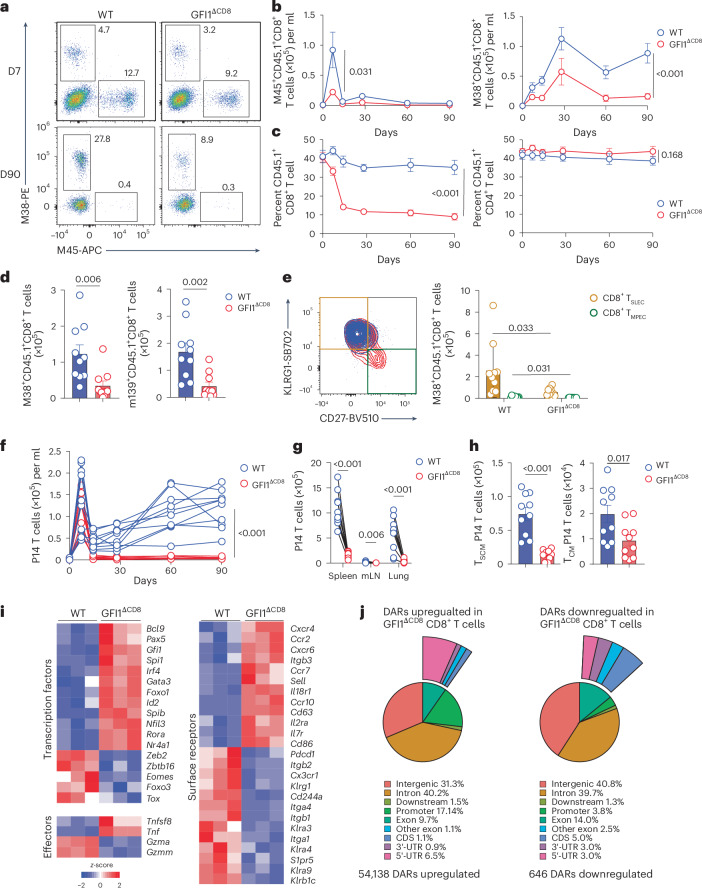
GFI1 is essential for persistent CD8^+^ T cell responses to cytomegalovirus infection. **a**,**b**, Representative flow cytometry plots at D7 and D90 (**a**) and total number at D0–D90 (**b**) of CD45.1^+^M45^+^ and CD45.1^+^M38^+^ CD8^+^ T cells post infection with MCMV in the peripheral blood of chimeric C57BL/6 mice that were reconstituted with a 1:1 mix of congenically labeled *Cd8a*^*cre*/+^ (WT) CD45.1^+^ and WT CD45.2^+^ (WT) or *Cd8a*^*cre*/+^*Gfi1*^fl/fl^ (GFI1^ΔCD8^) CD45.1^+^ and WT CD45.2^+^ (GFI1^ΔCD8^) bone marrow (BM) after lethal irradiation and infected with MCMV 8 weeks post BM reconstitution. **c**, Frequency of blood CD45.1^+^CD8^+^ T cells and CD45.1^+^CD4^+^ T cells among CD8^+^ and CD4^+^ T cells in WT and GFI1^ΔCD8^ mice as in **a**. **d**, Total splenic M38^+^CD45.1^+^CD8^+^ and m139^+^CD45.1^+^CD8^+^ T cells at D90 post infection with MCMV in WT and GFI1^ΔCD8^ mice as in **a**. **e**, Representative plot (left) and quantification (right) of CD45.1^+^KLRG1^+^CD27^−^ CD8^+^ T_SLEC_ cells and CD45.1^+^KLRG1^−^CD27^+^ CD8^+^ T_MPEC_ cells in spleen at D90 post infection with MCMV in WT and GFI1^ΔCD8^ mice as in **a**. **f**, Kinetics of WT or GFI1^ΔCD8^ CD8^+^ P14 T cells at D0, D14, D30, D60 and D90 post MCMV-ie2-gp33 infection in the peripheral blood of C57BL/6 mice that received 1:1 mix of congenically labeled WT and GFI1^ΔCD8^ CD8^+^ P14 T_N_ cells followed by infection with MCMV-ie2-gp33 24 h post transfer. **g**, Number of WT and GFI1^ΔCD8^ CD8^+^ T cells in the spleen, mLN and lung at D90 post infection with MCMV-ie2-gp33 as in **f**. **h**, Number of CD8^+^ T_SCM_ and CD8^+^ T_CM_ cells in the spleen at D90 post infection with MCMV-ie2-gp33 as in **f**. Data are shown as means; error bars, s.e.m. **i**, Normalized gene expression in splenic WT and GFI1^ΔCD8^ CD8^+^ P14 T cells at D7 post infection with MCMV-ie2-gp33 as in **f**. **j**, Frequency of upregulated and downregulated DARs in splenic GFI1^ΔCD8^ CD8^+^ P14 T cells at D7 post infection with MCMV-ie2-gp33 as in **f**. Outer donut, distribution of DARs within the exonic regions (3′-UTR, 5′-UTR, CDS and other exons). *P* values: two-tailed Student’s *t*-test (**b**–**e**); two-tailed paired *t-*test (**f**–**h**). Data in **b**–**e** are pooled from three experiments; mean values are shown; error bars, s.e.m. (*n* = 10 mice per group); data in **f**–**h** are pooled from two experiments (*n* = 10 mice). CDS, coding DNA sequence; UTR, untranslated region.

**Extended Data Fig. 7:**
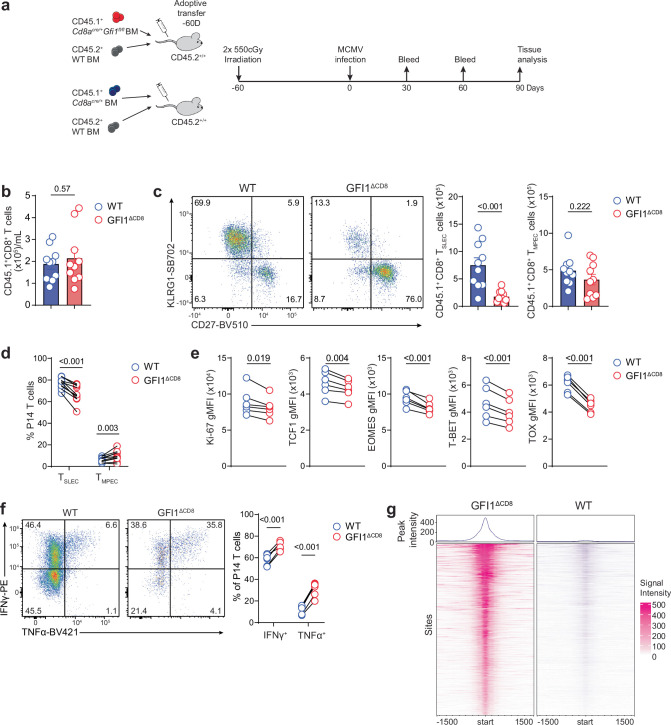
CD8^+^ T cell responses mounted by GFI1-deficient following latent MCMV infection. **a**, Schematic showing irradiation, BM cells injection, MCMV infection, peripheral blood and tissue analysis. **b**,**c**, Mixed bone marrow chimeras were generated by adoptive transfer of equal mix of WT CD45.1^+^ and WT CD45.2^+^ (WT + WT) or GFI1^ΔCD8^ CD45.1^+^ and WT CD45.2^+^ (WT + GFI1^ΔCD8^) bone marrow cells into irradiated C57BL/6 mice that were infected with MCMV intraperitoneally 60 days after reconstitution. **b**, Total number of CD45.1^+^CD8^+^ T cells in peripheral blood 8 weeks following bone marrow reconstitution. **c**, Flow cytometric dot plots showing frequency of T_SLEC_ (KLRG1^+^CD27^-^) and T_MPEC_ (KLRG1^-^CD27^+^) among activated (CD11a^+^CD44^+^) CD8^+^ T cells isolated from the spleen of bone marrow chimeric mice at D90 after infection (left panels). Total number of T_SLEC_ and T_MPEC_ CD45.1^+^CD8^+^ T cells in spleen at D90 (right panels). **b**, **c** Data pooled from 2 experiments (*n* = 9 or mice/condition) is shown as mean ± s.e.m. *p* values were calculated using a two-tailed unpaired Student’s *t* test. **d**,**e**, Naïve congenically-labelled WT (CD45.1^+^) and GFI1^ΔCD8^ (CD45.1^+^CD45.2^+^) P14 T cells were mixed 1:1 and co-transferred to C57BL/6 mice that were subsequently infected with MCMV-ie2-gp33 the next day. Mice were euthanized at D7 after infection to assess CD8^+^ T cell responses in tissues. **d**, Frequency of T_SELC_ and T_MPEC_ among WT and GFI1^ΔCD8^ P14 CD8^+^ T cells isolated from spleen at D7 after infection. Data pooled from two experiments (*n* = 10 mice). **e**, Data show Ki-67, TCF1, EOMES, T-BET and TOX expression from one of two independent experiments (*n* = 6 mice). **f**, Frequency of IFN-γ^+^ and IFN-γ^+^TNFα^+^ among total WT and GFI1^ΔCD8^ P14 CD8^+^ T cells isolated from spleen following gp33 peptide stimulation (left panels). Data showing frequency of IFN-γ^+^ and TNFα^+^ P14 CD8^+^ T cells from one of two independent experiments (*n* = 5 mice). **d**-**f**, *p* values calculated using a two-tailed paired *t* test. **g**, Chromatin accessibility read density enrichment around peak centres for adoptively transferred congenically-labelled WT or GFI1^ΔCD8^ P14 CD8^+^ T cells (CD45.1^+^) in response to MCMV-ie2-gp33 at D7. Upper panels show the average profile around detected peak centres. Lower panels show read intensity heatmaps ranked by total read intensity. Reads are centred on the middle of the accessible peak ±1.5 Kbp. Data pooled from three samples to generate read density map.

Murine cytomegalovirus (MCMV) infection is characterized by the development of ‘memory inflation’ driven by T_SCM_ cell precursors^6,30,31^. To understand the role of GFI1 in the development of these unusual T cell populations, we generated mixed bone marrow chimeras with WT or GFI1^ΔCD8^ bone marrow cells. Then, 8 weeks after bone marrow reconstitution, mice were infected with MCMV, and antigen-specific CD8^+^ T cells were monitored longitudinally in blood (Extended Data Fig. 7a). The number of WT and GFI1^ΔCD8^ CD8^+^ T cells in peripheral blood of chimeric mice was similar before virus infection (Extended Data Fig. 7b), suggesting that CD8^+^ T_N_ cell development from GFI1^ΔCD8^ bone marrow was not impaired. Following MCMV infection, WT CD8^+^ T cells mounted a strong response to the non-inflationary M45 epitope at day 7, followed by a sharp contraction of the response (Fig. 4a,b). No change in CD4^+^ T cell frequency was observed (Fig. 4c). WT M38-specific CD8^+^ T cells accumulated gradually to produce ‘memory inflation’ during virus latency (Fig. 4a,b)^31,32^. By contrast, GFI1^ΔCD8^ CD8^+^ T cells had impaired responses for both epitopes and contracted prematurely (Fig. 4a,b). The M38 and m139 inflationary epitope-specific GFI1^ΔCD8^ CD8^+^ T cells were significantly lower in spleen during latency (Fig. 4d). The inflationary T cell responses have been previously found to be maintained by continuous production of KLRG1^+^CD27^−^ CD8^+^ T_SLEC_ cells, which arise from KLRG1^−^CD27^+^ memory precursor CD8^+^ T (T_MPEC_) cells^30,31^. The GFI1^ΔCD8^ M38-specific and total activated CD11a^+^CD44^+^CD8^+^ T cell population exhibited a lower frequency of CD8^+^ T_SLEC_ cells (Fig. 4e and Extended Data Fig. 7c). Thus, GFI1 deficiency resulted in loss of long-term CD8^+^ T cell responses, in particular inflationary responses, following latent virus infection. GFI1^ΔCD8^ P14 CD8^+^ T cells mounted an impaired inflationary response following infection with a recombinant MCMV that expressed LCMV gp33 epitope (MCMV-ie2-gp33) (Fig. 4f,g). This included reduced CD8^+^ T_SCM_ cells, CD8^+^ T_CM_ cells (Fig. 4h) and CD8^+^ T_SLEC_ cells (Extended Data Fig. 7d) and lower Ki-67, TCF1, EOMES and TOX expression (Extended Data Fig. 7e). Altogether, these data demonstrate the essential role of GFI1 in mounting inflationary CD8^+^ T cell responses in MCMV infection.

Transcriptional analysis showed that GFI1^ΔCD8^ CD8^+^ T cells downregulated T_EFF_ cell surface receptor genes such as *Cx3cr1* and *Klrg1* (Fig. 4i and Supplementary Table 8). Furthermore, the transcriptional profile of GFI1^ΔCD8^ P14 CD8^+^ T cells was similar following infection with either MCMV-ie2-gp33 or chronic LCMV^c13^ (Fig. 3i), with GFI1^ΔCD8^ cells showing both *Eomes* and *Tox* downregulation (Fig. 4i). We observed that *Tnf* expression was upregulated in GFI1^ΔCD8^ P14 CD8^+^ T cells (Fig. 4i). Peptide stimulation confirmed a higher frequency of IFNγ^+^ and TNFα^+^ cells among GFI1^ΔCD8^ CD8^+^ T cells compared to WT CD8^+^ T cells (Extended Data Fig. 7f). Following MCMV infection, ATAC-seq revealed that GFI1 deficiency resulted in increased chromatin accessibility in GFI1^ΔCD8^ CD8^+^ T cells (Fig. 4j and Extended Data Fig. 7g), with 54,137 upregulated DARs and 646 downregulated DARs (Supplementary Table 9) compared to WT CD8^+^ T cells. Thus, GFI1 acted to epigenetically repress the transcriptional landscape of CD8^+^ T cells and promote inflationary T cell responses to cytomegalovirus infection.

### GFI1 epigenetically regulates T_SCM_ CD8^+^ T cell transcription

**Fig. 5:**
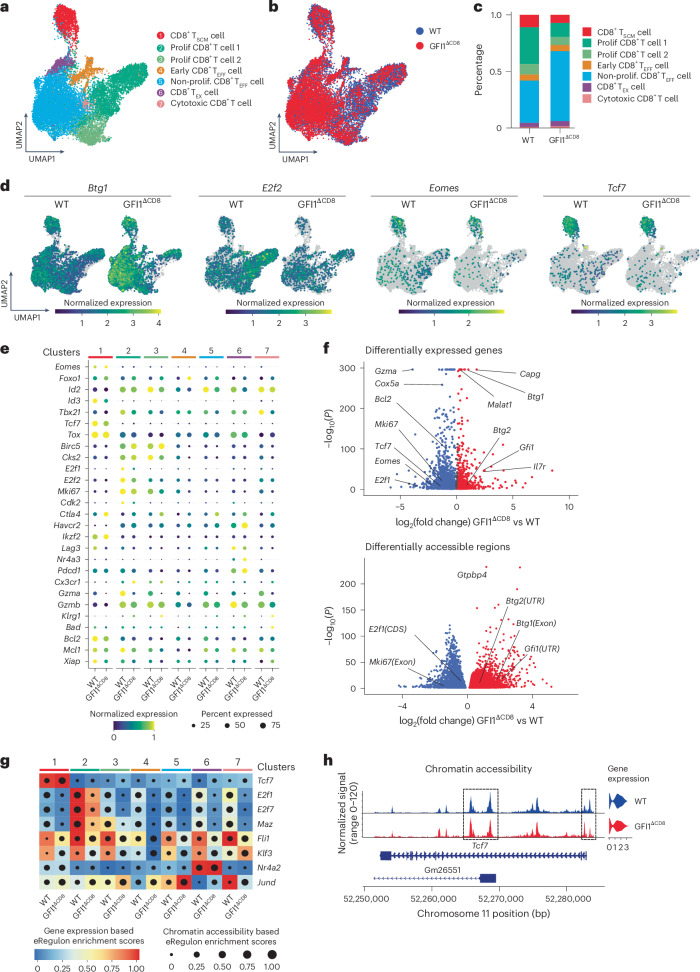
scMultiome-seq delineates GFI1-mediated epigenetic and transcriptional regulation in virus-specific CD8^+^ T cells following infection with LCMV^c13^. **a**, UMAP showing unsupervised clustering of 7,629 WT and 5,805 GFI1^ΔCD8^ CD8^+^ T cells isolated at D7 post infection with LCMV^c13^ from the spleen of C57BL/6 mice transferred i.v. with congenically labeled WT or GFI1^ΔCD8^ CD8^+^ T_N_ cells 24 h before infection, analyzed using scMultiome-seq (scRNA-seq + scATAC-seq Seurat-integrated data). **b**, UMAP showing WT and GFI1^ΔCD8^ CD8^+^ P14 T cell distribution in integrated data clusters as in **a**. **c**, Percentage of WT and GFI1^ΔCD8^ CD8^+^ P14 T cells in each cluster as in **a**. **d**, UMAP showing normalized expression of *Btg1, E2f2, Eomes* and *Tcf7* in WT and GFI1^ΔCD8^ CD8^+^ P14 T cells as in **a**. **e**, Dot plot showing expression of selected genes in WT and GFI1^ΔCD8^ CD8^+^ P14 T cells in clusters 1–7 as in **a**. Dot size indicates fraction of cells expressing gene; color represents mean expression. **f**, DEGs (top) and DARs (bottom) from cluster 1 as in **a**, using pseudobulk analysis of scRNA-seq and scATAC-seq, respectively. **g**, Selected top-ranking transcription-factor-linked eRegulons predicted by SCENIC+ analysis using scRNA-seq and scATAC-seq as in **a**. Color scale shows gene expression-based enrichment score; dot size illustrates chromatin accessibility-based enrichment score for each eRegulon and cell cluster. **h**, WT and GFI1^ΔCD8^ CD8^+^ T cell chromatin accessibility and gene expression at *Tcf7* gene locus in cluster 1 as in **a**. Dashed boxes highlight differentially accessible chromatin regions.

**Extended Data Fig. 8:**
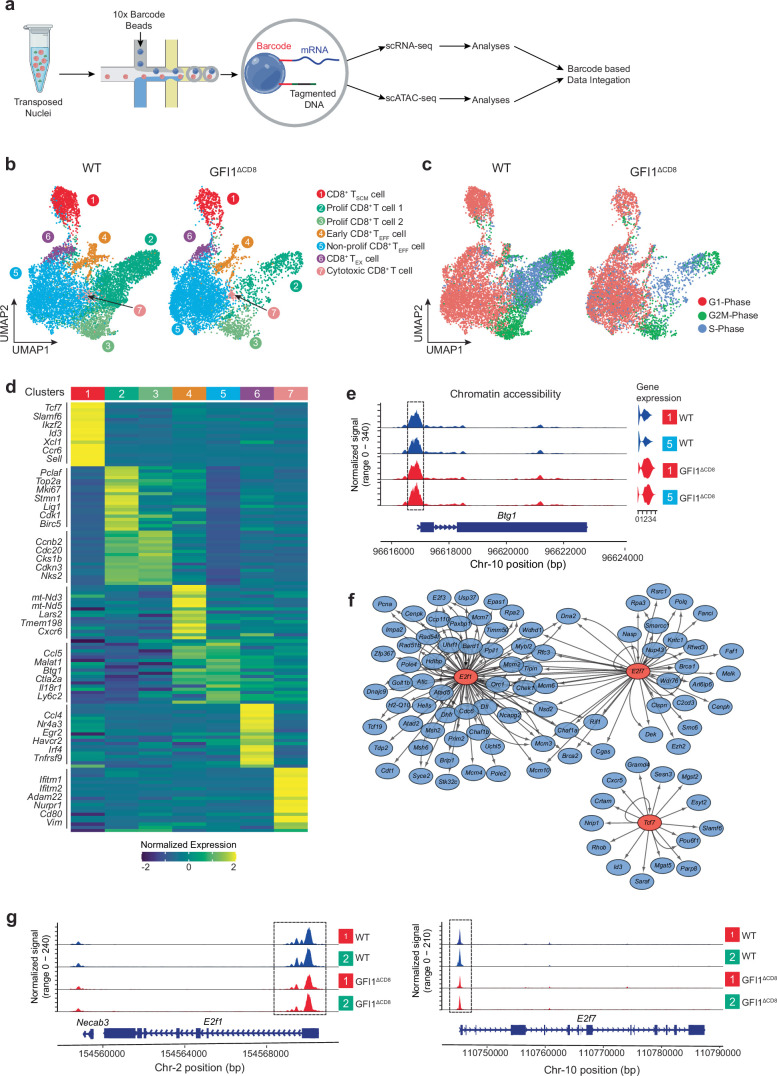
scMultiome-seq analysis of virus-specific CD8^+^ T cells following chronic virus infection. Naïve congenically-labelled WT or GFI1^ΔCD8^ P14 CD8^+^ T cells (CD45.1^+^) were transferred to C57BL/6 mice that were subsequently infected with LCMV^c13^ the next day. P14 T cells were isolated on D7 after infection for scMutiomic-seq. **a**, Schematics showing scMutiomic-seq workflow. **b**, UMAP projection showing unsupervised clustering of WT and GFI1^ΔCD8^ CD8^+^ T cells (WT + GFI1^ΔCD8^ Seurat integrated data). **c**, UMAP projection showing cell-cycle phases of WT and GFI1^ΔCD8^ CD8^+^ T cells based on expression of G2/M and S phase genes (scRNA-seq data). **d**, Aggregate gene expression of top 20 upregulated genes for each cluster. **e**, Cluster 1 and 5 WT (blue histogram and violin plots) and GFI1^ΔCD8^ (red histogram and violin plots) CD8^+^ T cell chromatin accessibility and gene expression at *Btg1* gene locus. Dashed boxes highlight differentially assessable chromatin regions. **f**, Transcription factor linked gene regulatory networks identified by SCENIC+ analysis. Core regulatory genes shown in red and target genes in blue. **g**, Cluster 1 and 2 WT (blue histogram and violin plots) and GFI1^ΔCD8^ (red histogram and violin plots) CD8^+^ T cell chromatin accessibility at *E2f1* (left panel) and *E2f7* (right panel) gene locus. Dashed boxes highlight differentially assessable chromatin regions.

To map the epigenetic and transcriptional landscape at single-cell level, we performed single-cell multiome sequencing (scMultiome-seq), which combines ATAC and gene expression analyses on WT and GFI1^ΔCD8^ P14 CD8^+^ T cells isolated at day 7 after LCMV^c13^ infection (Extended Data Fig. 8a). Uniform manifold approximation and projection (UMAP) and unsupervised clustering of integrated WT and GFI1^ΔCD8^ CD8^+^ T cells divided them into seven clusters based on their epigenome and transcriptome (Fig. 5a,b and Extended Data Fig. 8b). Cluster 1 was identified as CD8^+^ T_SCM_ cell precursors and clusters 2 and 3 were proliferating cells, respectively (Extended Data Fig. 8c). Cluster 1 cells expressed high levels of *Tcf7*, *Slamf6* and *Id3* and were thus annotated as CD8^+^ T_SCM_ cells (Extended Data Fig. 8d), while cluster 2 and cluster 3 cells expressed high levels of *Cdk1*, *Birc5* and *Mki67* (Extended Data Fig. 8d). The GFI1^ΔCD8^ CD8^+^ T cell population had fewer cells in clusters 1 and 2 (Fig. 5c and Extended Data Fig. 8b). By contrast, a higher fraction of the GFI1^ΔCD8^ CD8^+^ T population was located in cluster 5 (Fig. 5c). This population exhibited reduced expression of genes associated with proliferation and high expression of *Btg1* (Extended Data Fig. 8d). GFI1^ΔCD8^ CD8^+^ T cells had high *Btg1*, while *E2f2*, *Eomes* and *Tcf7* was downregulated (Fig. 5d,e). scATAC-seq data confirmed increased chromatin accessibility at the *Btg1* locus in clusters 1 and 5 (Extended Data Fig. 8e). To identify DEGs and DARs within the CD8^+^ T_SCM_ cell cluster, we performed pseudobulk RNA-seq (Supplementary Table 10) and ATAC-seq (Supplementary Table 11) analyses on cluster 1 cells. These analyses showed that loss of GFI1 led to increased *Btg1* and *Btg2* expression, whereas the expression of *Tcf7*, *Eomes*, *Bcl2* and *Mki67* was reduced in CD8^+^ T_SCM_ cells (Fig. 5f). These transcriptional changes were linked to upregulated DARs in the *Btg1* and *Btg2* gene loci and downregulated DARs in *E2f1* and *Mki67* genes (Fig. 5f). Thus, GFI1^ΔCD8^ CD8^+^ T_SCM_ cells had reduced proliferation gene expression and increased quiescence gene expression.

To further understand the gene regulatory networks of each cluster, SCENIC+ analysis^33^ identified enhancer-driven gene regulatory networks and candidate upstream transcription factors for each cell cluster (Fig. 5g). The top-ranked eRegulon for cluster 1 was regulated by *Tcf7* (Fig. 5 and Extended Data Fig. 8f), in line with the high expression of memory-related transcription factors such as *Eomes*, *Id3* and *Myb* within that cluster. The chromatin region enrichment score of *Tcf7*-driven eRegulon was higher for cluster 1 GFI1^ΔCD8^ CD8^+^ T cells owing to upregulated DARs in the *Tcf7* gene body, while the chromatin accessibility at the 5′-untranslated region of *Tcf7* was reduced (Fig. 5h), in agreement with reduced *Tcf7* expression in GFI1^ΔCD8^ CD8^+^ T cells (Fig. 5e). Additionally, gene regulatory network analyses identified that *E2f1*-driven and *E2f7*-driven direct gene networks important for regulating cell proliferation were disrupted in GFI1^ΔCD8^ CD8^+^ T cells (Fig. 5g and Extended Data Fig. 8g). These findings suggest that GFI1 is a key epigenetic modulator of transcriptional networks critical for memory CD8^+^ T cell proliferation.

### GFI1 enhances CD8^+^ T cell recall responses and survival

**Fig. 6:**
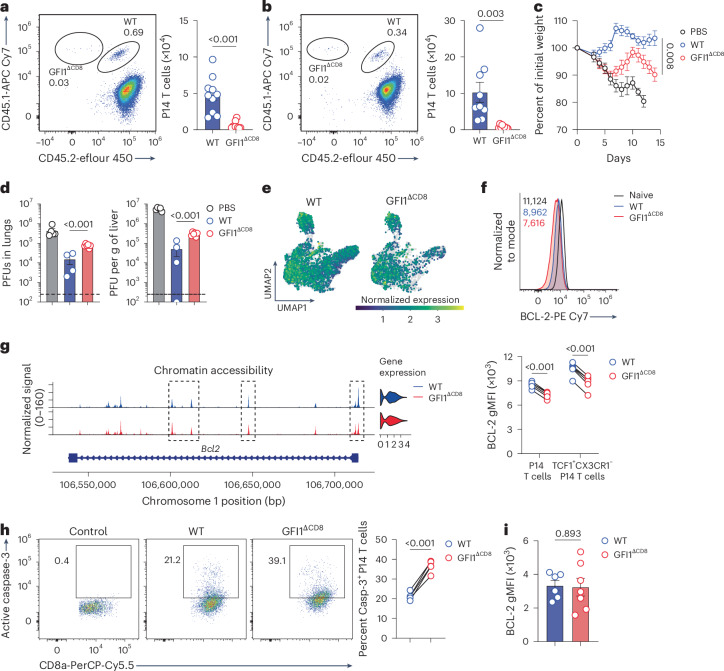
GFI1 is required for secondary CD8^+^ T cell responses. **a**,**b**, Representative plot (left) and quantification (right) of donor WT and GFI1^ΔCD8^ CD8^+^ T cells at D7 post infection with LCMV^Arm^ (**a**) or MCMV-ie2-gp33 (**b**) in the spleen of secondary C57BL/6 recipients transferred with 1:1 mix of congenically labeled activated WT and GFI1^ΔCD8^ CD8^+^ T cells isolated at D7 post infection with LCMV^c13^ from the spleen of primary C57BL/6 recipients that received congenically labeled WT or GFI1^ΔCD8^ CD8^+^ T_N_ cells 24 h before infection with LCMV^c13^. Data are pooled from two experiments (*n* = 9 or 10 mice); mean values are shown; error bars, s.e.m. **c**, Weight-loss kinetics of *Rag2*^–/–^*Il2rγ*^–/–^ mice that received activated WT or GFI1^ΔCD8^ CD8^+^ P14 T cells from C57BL/6 mice that received WT or GFI1^ΔCD8^ CD8^+^ P14 T_N_ cells 1 day before infection with LCMV^c13^. **d**, Virus titer in the lungs and liver of *Rag2*^–/–^*Il2rγ*^–/–^ at D14 post transfer of activated WT or GFI1^ΔCD8^ CD8^+^ P14 T cells as in **c**. Dashed line, assay detection limit. **e**, scMultiome-seq data showing expression of *Bcl2* in splenic WT and GFI1^ΔCD8^ CD8^+^ T cells isolated at D7 post infection from C57BL/6 mice that received congenically labeled WT or GFI1^ΔCD8^ CD8^+^ T_N_ cells 24 h before LCMV^c13^ infection. **f**, Representative histogram (top) and quantification (bottom) of BCL-2 expression at D7 in splenic CD8^+^ P14 T cells isolated from C57BL/6 mice transferred with a 1:1 mix of congenically labeled WT and GFI1^ΔCD8^ T_N_ cells 24 h before LCMV^c13^ infection. Data are representative of two experiments (*n* = 6 mice). **g**, Cluster 1 WT (blue) and GFI1^ΔCD8^ (red) CD8^+^ T cell chromatin accessibility and gene expression at the *Bcl2* locus as in **e**. Dashed boxes, DARs. **h**, Representative plots (left) and quantification (right) of splenic WT and GFI1^ΔCD8^ caspase-3^+^CD8^+^ T cells at D7 post LCMV^c13^ infection as in **f**. Control, FMO staining of WT CD8^+^ T cells. Data are representative of two experiments (*n* = 5 mice). **i**, BCL-2 expression in CD8^+^ T_N_ cells from the spleen of naive WT and GFI1^ΔCD8^ mice; mean ± s.e.m. Data are pooled from two experiments (*n* = 6 or 7 mice per genotype). *P* values were calculated using a two-tailed paired *t*-test (**a**, **b**, **f** and **h**) or two-tailed Student’s *t*-test (**c**, **d** and **i**). Data in **c** and **d** are representative of two experiments; mean values are shown; error bars, s.e.m. (*n* = 4, 5 or 6 mice per condition).

**Extended Data Fig. 9:**
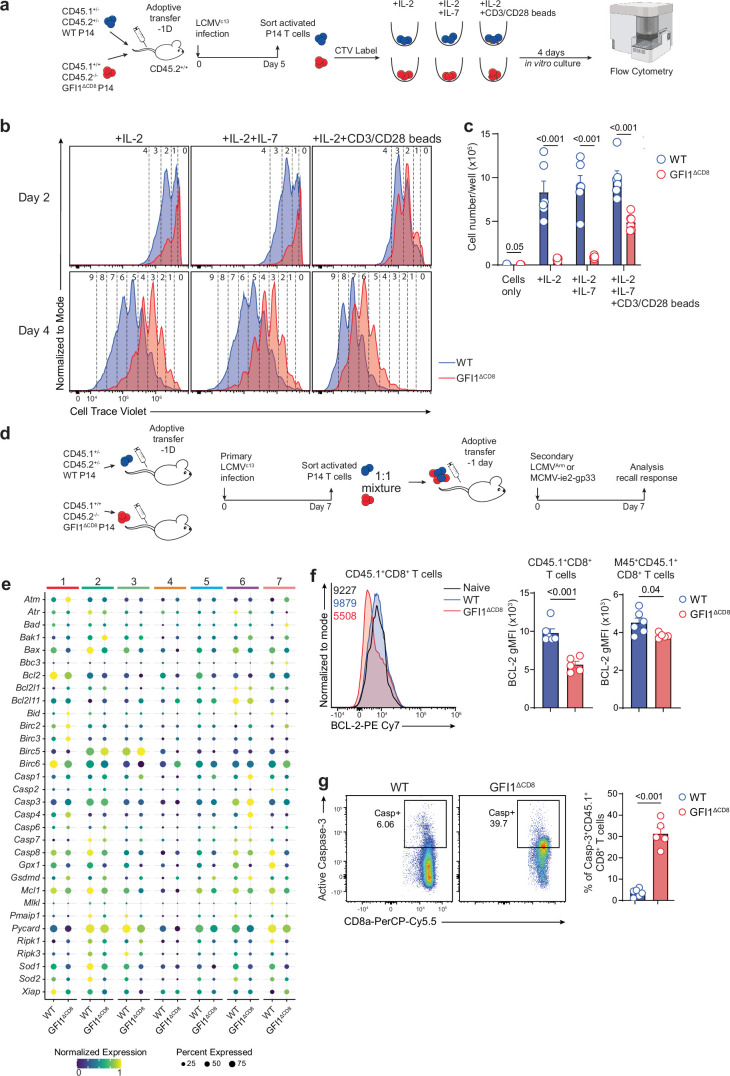
GFI1-deficient CD8^+^ T cells exhibit reduced proliferation and are prone to activation induced T cell death. **a**, Schematic representation showing naïve P14 CD8^+^ T cells transfer to C57BL/6 mice that were infected with LCMV^c13^ after 24 h. P14 T cells were isolated at D5 after infection, labelled with CTV then cultured *in vitro* for 4 days with IL-2, IL-2 + IL-7 or IL-2 + CD3/CD28 beads. **b**, Flow cytometric histograms showing WT (blue) and GFI1^ΔCD8^ (red) CD8^+^ T cell proliferation. Dotted lines mark cell divisions. **c**, Total number of WT and GFI1^ΔCD8^ CD8^+^ T cells per well after 4 days *in vitro* culture shown as mean ± s.e.m. Data pooled from two independent experiments (*n* = 6 mice). *p* values were calculated using a two-tailed Student’s *t* test. **d**, Schematics showing P14 T cell activation with primary infection, sorting, and transfer to secondary recipients and virus infection. **e**, Naïve congenically-labelled WT or GFI1^ΔCD8^ P14 CD8^+^ T cells (CD45.1^+^) were transferred to C57BL/6 mice that were subsequently infected with LCMV^c13^ the next day. P14 T cells were isolated on D7 after infection for scMutiomic-seq. Dot plot showing expression of selected cell death related genes in WT and GFI1^ΔCD8^ P14 T cells, with dot size indicating fraction of cells expressing gene and colour representing mean expression. **f**, Mixed bone marrow chimeras were generated by adoptive transfer of equal mix of WT CD45.1^+^ and WT CD45.2^+^ (WT + WT) or GFI1^ΔCD8^ CD45.1^+^ and WT CD45.2^+^ (WT + GFI1^ΔCD8^) bone marrow into irradiated C57BL/6 mice that were infected with MCMV intraperitoneally at D60 after reconstitution. Histograms (left panel) and bar plots (right panels) showing expression of BCL-2 in CD8^+^ T cells isolated from spleen at D7 after infection shown as mean ± s.e.m. **g**, Flow cytometric plot showing frequency of caspase-3^+^ (casp-3) CD45.1^+^CD8^+^ T cells isolated from spleen 7 days after infection (left panels). Frequency of casp-3^+^ CD45.1^+^ CD8^+^ T cells in spleen 7 days after infection shown as mean ± s.e.m. (right panel). **f, g**, Data pooled from two experiments (*n* = 5 or 6mice). *p* values were determined using a two-tailed unpaired Student’s *t* test.

Given the changes in gene expression affecting cell proliferation, TCR and cytokine-mediated proliferation of GFI1^ΔCD8^ CD8^+^ T_M_ cells, isolated from spleen of LCMV^c13^-infected mice at day 7 after infection, was quantitated by culturing CD8^+^ T_M_ cells in vitro with IL-2 + IL-7 or IL-2 + CD3/CD28 stimulation beads (Extended Data Fig. 9a). This showed that fewer GFI1^ΔCD8^ CD8^+^ T cells entered division when exposed to cytokines IL-2 + IL-7 and exhibited extremely limited expansion compared with WT CD8^+^ T cells (Extended Data Fig. 9b,c). Although TCR activation was able to drive strong proliferation by day 4 post-stimulation in all conditions, GFI1^ΔCD8^ CD8^+^ T cells showed significantly less proliferation than WT CD8^+^ T cells (Extended Data Fig. 9b,c). To investigate whether the impaired proliferation of GFI1^ΔCD8^ CD8^+^ T cells observed following primary infection also impacted CD8^+^ T cell recall responses, we isolated WT and GFI1^ΔCD8^ CD8^+^ T_M_ cells from LCMV^c13^-infected mice at day 5 after infection and co-transferred them to a secondary host followed by heterologous infection with LCMV^Arm^ or MCMV-ie2-gp33 (Extended Data Fig. 9d). This procedure showed that recall responses by GFI1^ΔCD8^ CD8^+^ T_M_ cells were severely impaired (Fig. 6a,b). To test the antiviral capacity of GFI1^ΔCD8^ CD8^+^ T_M_ cells, WT or GFI1^ΔCD8^-activated P14 CD8^+^ T cells were transferred to *Rag2*^–/–^*Il2rγ*^–/–^ mice, which lack natural killer cells and CD4^+^ T cell responses to MCMV^34^. Although WT CD8^+^ T cells were able to mediate effective protection against MCMV infection, mice receiving GFI1^ΔCD8^ CD8^+^ T cells exhibited severe weight loss (Fig. 6c). This finding correlated with impaired virus control in the lungs and liver in mice reconstituted with GFI1^ΔCD8^ CD8^+^ T cells (Fig. 6d). These results demonstrate that GFI1 is crucial for recall responses and antiviral functions of memory CD8^+^ T cells.

scRNA-seq of spleen CD8^+^ T_M_ cells isolated day 7 after LCMV^c13^ infection showed that anti-apoptotic genes such as *Bcl2*, *Mcl1* and *Xiap* were diminished, while pro-apoptotic genes like *Bax* and *Bid*^35,36^ were elevated in GFI1^ΔCD8^ CD8^+^ T cells compared to WT CD8^+^ T cells (Extended Data Fig. 9e). *Bcl2* transcript levels were notably lower in clusters 1 and 2 in GFI1^ΔCD8^ CD8^+^ T cells compared to WT CD8^+^ T cells (Fig. 6e and Extended Data Fig. 9e). This paralleled lower BCL-2 expression (Fig. 6f) and reduced accessibility at the *Bcl2* locus (Fig. 6g) in GFI1^ΔCD8^ CD8^+^ T_SCM_ cells. Elevated caspase-3 activation indicated increased apoptosis in GFI1^ΔCD8^ CD8^+^ T cells (Fig. 6h). Similar patterns were observed following MCMV infection (Extended Data Fig. 9f,g). However, GFI1^ΔCD8^ CD8^+^ T_N_ cells had normal BCL-2 expression (Fig. 6i), indicating the specific role of GFI1 in CD8^+^ T cell survival post activation and its action to inhibit activation-induced T cell death. Altogether, these data show that GFI1 promotes CD8^+^ T cell proliferation, survival and virus control capacity.

### EOMES rescues GFI1^ΔCD8^ CD8^+^ T cell persistence

**Fig. 7:**
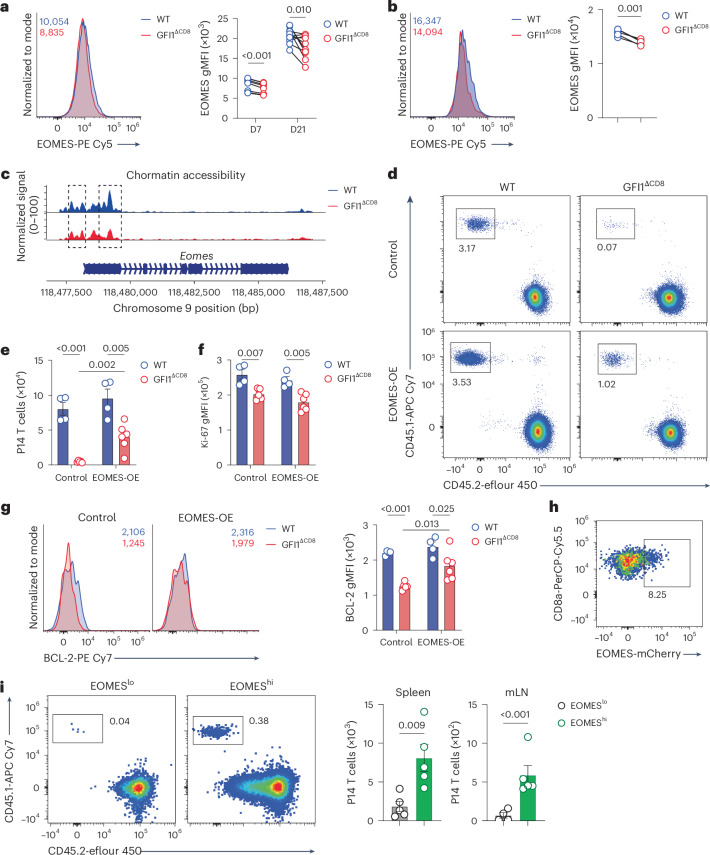
GFI1 epigenetically regulates EOMES expression in memory CD8^+^ T cells. **a**, Representative histogram (left) and quantification (right) of EOMES expression in splenic WT and GFI1^ΔCD8^ CD8^+^ P14 T cells at D7 post infection in C57BL/6 mice that received a 1:1 mix of congenically labeled WT and GFI1^ΔCD8^ CD8^+^ P14 T cells 24 h before infection with LCMV^c13^. Data are pooled from two experiments (*n* = 10 mice per time point). **b**, Representative histogram (left) and quantification (right) of EOMES expression in splenic WT and GFI1^ΔCD8^ CD8^+^ T_SCM_ cells at D7 post LCMV^c13^ infection as in **a**. One of two experiments is shown (*n* = 5 mice). **c**, Chromatin accessibility at *Eomes* locus in WT (blue) and GFI1^ΔCD8^ (red) CD8^+^ T cells isolated at D7 post infection from the spleen of C57BL/6 mice that received congenically labeled WT or GFI1^ΔCD8^ CD8^+^ T_N_ cells 24 h before infection with LCMV^c13^; analyzed with scMultiome-seq. Dashed boxes, DARs. **d,e** Representative plots (**d**) and quantification (**e**) of WT and GFI1^ΔCD8^ CD45.1^+^CD8^+^ P14 T cells at D14 in the spleen of CD45.2^+^ C57BL/6 mice transferred with WT or GFI1^ΔCD8^ CD45.1^+^CD8^+^ P14 T cells transduced with control or EOMES-expressing (EOMES-OE) lentivirus and infected with LCMV^c13^ 24 h later. **f**, Ki-67 expression in splenic WT and GFI1^ΔCD8^ CD8^+^ P14 T cells as in **d**. **g**, Representative histograms (left) and quantification (right) of BCL-2 expression in splenic WT and GFI1^ΔCD8^ CD8^+^ P14 T cells as in **d**. **h**, Representative flow cytometry of splenic EOMES^hi^CD8^+^ P14 T cells at D7 post infection in C57BL/6 mice that received congenically labeled *Eomes*^*mCherry*/+^ CD8^+^ P14 T_N_ cells 24 h before infection with LCMV^c13^. Data are representative of two independent experiments. **i**, Representative flow cytometry plot (left) and quantification (right) of splenic CD45.1^+^CD8^+^ P14 T cells at D21 post LCMV^c13^ infection in secondary infection recipients that at D7 post infection received matched (D7 post infection) activated CD45.1^+^EOMES^hi^ or CD45.1^+^EOMES^lo^ CD8^+^ T cells isolated from primary-infected C57BL/6 mice, which were adoptively transferred with *Eomes*^*mCherry*/+^ CD8^+^ P14 T_N_ cells 1 day before primary infection with LCMV^c13^. Data are from one experiment; mean values are shown; error bars, s.e.m. (*n* = 5 mice per condition). *P* values were calculated using a two-tailed paired *t*-test (**a**, **b** and **j**) or two-tailed Student’s *t*-test (**e**–**g**). Data in **d**–**g** are pooled from two experiments (*n* = 4 or 6 mice per condition); mean values are shown; error bars, s.e.m.

TCF1 promotes memory CD8^+^ T cell persistence through EOMES^37^. Given the downregulation of EOMES in CD8^+^ T_SCM_ cells (Fig. 7a,b) and reduced chromatin accessibility at the *Eomes* locus in GFI1^ΔCD8^ CD8^+^ T_M_ cells (Fig. 7c), we examined the requirement for GFI1-mediated EOMES in maintaining the persistence of CD8^+^ T cells following chronic viral infection. WT and GFI1^ΔCD8^ P14 CD8^+^ T cells transduced with *Eomes* were adoptively transferred into congenic recipient mice that were infected with LCMV^c13^. EOMES overexpression partially rescued the number of GFI1^ΔCD8^ CD8^+^ T cells (Fig. 7d,e) but did not impact proliferation, despite increased BCL-2 expression (Fig. 7f,g), suggesting that GFI1 drove CD8^+^ T cell survival partly by regulating EOMES but requires additional regulators to fully support proliferation.

Examination of P14 CD8^+^ T cells from *Eomes*^*mCherry*^ reporter mice revealed approximately 5–10% of CD8^+^ T cells had high expression of EOMES on day 7 after LCMV^c13^ primary infection (Fig. 7h). Adoptive transfer of these EOMES^hi^ T cells into infection-matched secondary recipients showed that these EOMES^hi^CD8^+^ T cells had higher re-population capacity than EOMES^lo^ CD8^+^ T cells (Fig. 7i). These results indicate that early emerging EOMES-expressing CD8^+^ T cells are crucial for long-term persistence during chronic infection, highlighting the importance of GFI1-driven EOMES expression for antiviral T cell memory.

### Continuous GFI1 expression maintains CD8^+^ T cell persistence

**Fig. 8:**
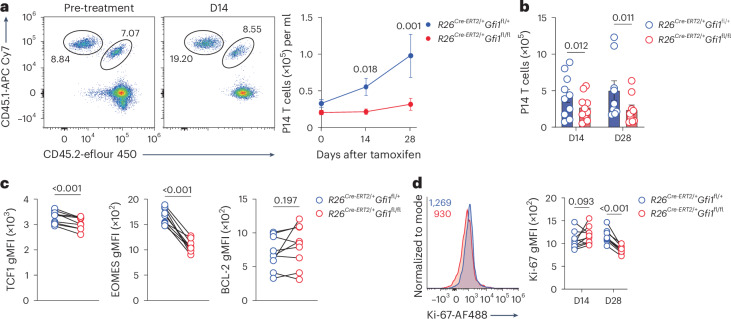
Continuous expression of GFI1 is required to maintain CD8^+^ T cell persistence following chronic viral infection. **a**, Representative flow cytometry plot (left) and quantification (right) of *R26*^*Cre-ERT2*/+^*Gfi1*^fl/+^ and *R26*^*Cre-ERT2*/+^*Gfi1*^fl/fl^ CD8^+^ P14 T cells in peripheral blood of MCMV-ie2-gp33-infected C57BL/6 mice that received 1:1 mix of congenically labeled *R26*^*Cre-ERT2/+*^*Gfi1*^fl/+^ and *R26*^*Cre-ERT2*/+^*Gfi1*^fl/fl^ CD8^+^ P14 T cells 30 days before tamoxifen treatment and were infected with MCMV-ie2-gp33 1 day post CD8^+^ T cell adoptive transfer; mean values are shown; error bars, s.e.m. **b**, Number of *R26*^*Cre-ERT2*/+^*Gfi1*^fl/+^ and *R26*^*Cre-ERT2*/+^*Gfi1*^fl/fl^ CD8^+^ P14 T cells in spleen at D14 and D28 after tamoxifen treatment as in **a**; mean values are shown; error bars, s.e.m. **c**, Expression of TCF1, EOMES and BCL-2 in *R26*^*Cre-ERT2*/+^*Gfi1*^fl/+^ and *R26*^*Cre-ERT2*/+^*Gfi1*^fl/fl^ CD8^+^ P14 T cells at D28 after tamoxifen treatment as in **a**. **d**, Representative histogram at D28 (right) and quantification at D14 and D28 (left) of Ki-67 expression in splenic *R26*^*Cre-ERT2*/+^*Gfi1*^fl/+^ and *R26*^*Cre-ERT2*/+^*Gfi1*^fl/fl^ CD8^+^ P14 T cells as in **a**. In **b**–**d**, data are pooled from two independent experiments (*n* = 9 or 10 mice per time point). *P* values were calculated using a two-tailed paired *t*-test.

**Extended Data Fig. 10:**
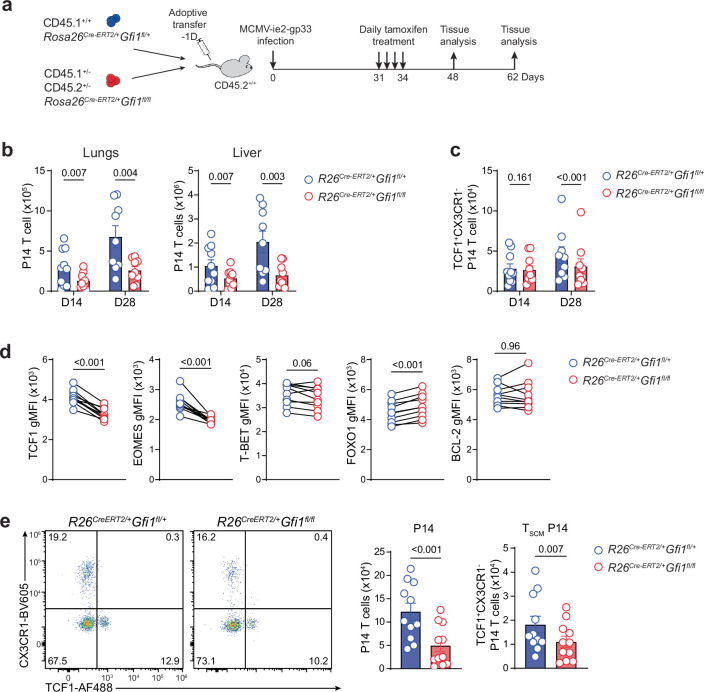
Continuous expression of GFI1 is required to maintain persistence of antiviral CD8^+^ T cells. **a**-**d**, Congenically-labelled naïve *R26*^*Cre-ERT2/+*^*Gfi1*^*fl/+*^ and *R26*^*Cre-ERT2/+*^*Gfi1*^*fl/fl*^ P14 T cells were co-transferred to C57BL/6 mice that were subsequently infected with MCMV-ie2-gp33 the next day. Mice were treated with tamoxifen daily for 4 days from day 30 after infection and CD8^+^ T cell responses analysed at D14 or D28 after tamoxifen. **a**, Schematic showing P14 T cell transfer, virus infection, tamoxifen treatment and analysis. **b**, Total number of P14 T cells in lungs and liver at D14 and D28 after tamoxifen treatment. **c**, Total number of T_SCM_ P14 T cells in spleen at D14 and D28 after tamoxifen treatment. **d**, Expression of TCF1, EOMES, T-BET, FOXO1 and BCL-2 in P14 T cells isolated from spleen of tamoxifen-treated animals at D14 after treatment. **b**-**d** Data pooled from two experiments/timepoint (*n* = 9 or 10 mice/timepoint). **e**, Congenically-labelled naïve *R26*^*Cre-ERT2/+*^*Gfi1*^*fl/+*^ and *R26*^*Cre-ERT2/+*^*Gfi1*^*fl/fl*^ P14 T cells were co-transferred to C57BL/6 mice that were subsequently infected with LCMV^c13^ the next day. Mice were treated with tamoxifen at D15 after infection for 4 days and CD8^+^ T cell responses were analysed on D30 after infection. Plot showing frequency of T_SCM_ P14 T cells in spleen at D30 (left panel). Total number of P14 and T_SCM_ T cells in spleen and lungs (left panel). Data pooled from two experiments (*n* = 11 mice). **b**-**e** Data show mean ± s.e.m. *p* values were determined using a two-tailed paired *t* test.

To assess whether GFI1 is essential for long-term CD8^+^ T cell maintenance after chronic infection, congenically labeled naive *R26*^*creERT2*/+^*Gfi1*^fl/+^ and *R26*^*creERT2*/+^*Gfi1*^fl/fl^ P14 CD8^+^ T cells were co-transferred into C57BL/6 recipients and infected with MCMV-ie2-gp33 virus (Extended Data Fig. 10a). Tamoxifen-induced GFI1 ablation resulted in a reduction of *R26*^*creERT2*/+^*Gfi1*^fl/fl^ CD8^+^ T cell numbers in blood and tissues compared to *R26*^*creERT2*/+^*Gfi1*^fl/+^ CD8^+^ T cells (Fig. 8a,b and Extended Data Fig. 10b), significantly decreasing the GFI1-deficient T_SCM_ cell population by day 28 (Extended Data Fig. 10c). TCF1, EOMES and T-BET expression were impaired at day 14 (Extended Data Fig. 10d) and remained low at day 28 (Fig. 8c), whereas BCL-2 levels were unchanged (Fig. 8c) in *R26*^*creERT2*/+^*Gfi1*^fl/fl^ CD8^+^ T cells. This was accompanied by reduced proliferation of *R26*^*creERT2*/+^*Gfi1*^fl/fl^ P14 CD8^+^ T cells at day 28 but not at day 14 (Fig. 8d). Similarly, tamoxifen-mediated GFI1 ablation in *R26*^*creERT2*/+^*Gfi1*^fl/fl^ at day 15 post LCMV^c13^ infection led to reduced *R26*^*creERT2*/+^*Gfi1*^fl/fl^ CD8^+^ T cell persistence (Extended Data Fig. 10e). Thus, continuous expression of GFI1 is crucial for sustaining persistent CD8^+^ T cell responses in chronic and latent infections.

## Discussion

Here, we defined a role of GFI1 in regulating CD8^+^ T_SCM_ cell persistence. CD8^+^ T_N_ cells had high expression of GFI1, and although GFI1 was rapidly downregulated in effector cells following activation, the CD8^+^ T_SCM_ cell population selectively maintained high GFI1 expression. GFI1 ablation markedly impaired T_M_ cells during chronic infection owing to reduced proliferation and increased GFI1-deficient CD8^+^ T cell death. Furthermore, we showed that continuous GFI1 expression was required to sustain CD8^+^ T cell proliferation during chronic infection. Collectively, these observations indicated that GFI1 maintained CD8^+^ T_SCM_ cell persistence by promoting proliferation and by inhibiting EOMES-dependent activation-induced cell death.

CD4^+^CD8^+^ (double-positive) thymic T cells express high levels of GFI1 (ref. ^22^), which evicts autoreactive T cells from the thymus to induce tolerance^38^. CD8^+^ T cells have been reported to transiently upregulate GFI1 after in vitro stimulation with concanavalin A^22^. We found that GFI1 was downregulated in CD8^+^ T_EFF_ cells following chronic infection but was maintained in memory subsets, including CD8^+^ T_SCM_ and CD8^+^ T_CM_ cells. Mapping GFI1 expression amongst CD8^+^ T_PEX_ populations showed that CD62L^+^CD8^+^ T_PEX_ cells maintained higher proliferation capacity^23^ and exhibited higher GFI1 than CD62L^−^CD8^+^ T_PEX_ cells, suggesting that GFI1 allowed CD62L^+^CD8^+^ T_PEX_ cells to retain proliferation characteristics.

GFI1 was found to be crucial for maintaining CD8^+^ T cell persistence during chronic viral infection. This lack of CD8^+^ T cell persistence could be attributed to reduced proliferation of GFI1-deficient CD8^+^ T_SCM_ cells, especially cytokine-driven proliferation. We observed impaired cell cycle regulation in GFI1-deficient CD8^+^ T_SCM_ cells, consistent with a previous observation that GFI1 is required for thymic T cell proliferation during the development^17^ and proliferation of other cell types, including hematopoietic stem cells^39^. GFI1 maintained the proliferation of CD8^+^ T_SCM_ cells by promoting gene regulatory networks driven by the E2F family, notably *E2f1* and *E2f7*. HOMER analysis showed a GFI1 binding motif in the promotor of *E2f7*, suggesting direct epigenetic regulation. Moreover, GFI1 promoted CD8^+^ T_SCM_ cell persistence by epigenetically silencing the expression of *Btg1* and *Btg2*, which has been shown to inhibit T cell quiescence^40^. We found that continuous expression of GFI1 was necessary to maintain CD8^+^ T_SCM_ cell proliferation, indicating that stable GFI1 expression in T_SCM_ cells was required to sustain the cell cycle regulatory circuits necessary for memory CD8^+^ T cell proliferation.

Transcriptional mapping showed that GFI1 loss impaired the expression of the transcriptional regulators *Id2* and *Zeb2*, which are important for T_EFF_ cell differentiation and CD8^+^ T_SLEC_ cell formation^25,41^. Reduced proliferation of CD8^+^ T_CM_ cells during latent MCMV infection was shown to impair CD8^+^ T_SLEC_ cell development, resulting in loss of MCMV-specific memory inflation^30^. Here, the impaired proliferation and subsequent loss of CD8^+^ T_SCM_ cells in the absence of GFI1 provides an explanation for the impaired MCMV-specific memory inflation in the GFI1-deficient CD8^+^ T cells. This highlights the requirement for GFI1 expression in CD8^+^ T cells throughout the course of chronic viral infection to generate immune protection.

We showed that GFI1 promoted BCL-2 expression in CD8^+^ T_SCM_ cells in an EOMES-dependent manner. Although GFI1 loss in CD8^+^ T_N_ cells did not alter EOMES or BCL-2 expression, GFI1-deficient CD8^+^ T_SCM_ cells exhibited reduced BCL-2. This reduction paralleled reduced chromatin accessibility at the *Bcl2* locus in CD8^+^ T_SCM_ cells and elevated death in these cells. This GFI1-mediated BCL-2 expression in CD8^+^ T_SCM_ cells is consistent with previous reports that BCL-2 overexpression protects GFI1-deficient thymic T cells from apoptosis^17,42^. The loss of GFI1 in CD8^+^ T_N_ cells did not impact the chromatin accessibility, but it altered the epigenetic profile of activated CD8^+^ T cells, indicating that GFI1 regulated CD8^+^ T cell chromatin accessibility following T cell activation. Thus, GFI1-mediated epigenetic gene regulation of CD8^+^ T_SCM_ cells promotes BCL-2 expression following CD8^+^ T cell activation but not in CD8^+^ T_N_ cells. The transcription factor EOMES was dysregulated in activated T cells following GFI1 ablation. EOMES inhibits activation-induced T cell death by promoting BCL-2 expression^37,43^. Overexpression of EOMES induced BCL-2 expression and partially rescued GFI1-deficient CD8^+^ T cell persistence. Impaired BCL-2 expression has also been reported in TCF1-deficient CD8^+^ T cells, and similar to GFI1-deficient CD8^+^ T cells, this phenotype was rescued by EOMES overexpression^37^. A binding motif for GFI1 in the *Eomes* promotor suggests that GFI1 directly regulates EOMES expression to support T_M_ cell but not T_N_ cell survival. Collectively, these findings demonstrate that GFI1 drives EOMES-dependent BCL-2 expression in CD8^+^ T cells to generate ‘fit’ T_M_ cells.

In conclusion, this study reveals an important function for GFI1 in maintaining CD8^+^ T cell persistence following chronic viral infection. We demonstrate that GFI1 is a key regulator of memory CD8^+^ T cells that sculpts their gene regulatory network by modulating epigenetic repression. Finally, we demonstrate that selective GFI1 expression in CD8^+^ T_SCM_ cells ensures their long-term persistence by promoting enduring proliferative potential required for self-renewal and maintenance of memory populations.

## Methods

### Mice

C57BL/6 (CD45.2^+/+^), B6.SJL-*Ptprc*^*a*^*Pep3*^*b*^/BoyJ (CD45.1^+/+^), *Gfi1*^*tdTomato*^^/+^ (refs. ^20,21^), *Eomes*^*mCherry*/+^ (ref. ^44^), *Rag2*^–/–^*Il2rγ*^–/–^, B6.Tg(Cd8a-cre)1Itan (*Cd8a*^*cre*/+^)^28^, B6.129-*Gt(ROSA)26Sor*^*tm1(cre/ERT2)Tyj*^/J (ref. ^45^) (*R26*^*cre/ERT2*/+^) and *Gfi1*^fl/fl^ (ref. ^46^) mice have been described previously. B6.Cg-*Tcra*^*tm1Mom*^Tg(TcrLCMV)327Sdz/TacMmjax (P14) mice carry CD8^+^ P14 T cells transgenic for the T cell antigen receptor specific for the LCMV-derived gp33-41 epitope^47^. *Gfi1*^*tdTomato*/+^ mice were backcrossed to the C57BL/6 background for at least ten generations. *Gfi1*^fl/fl^ and *Eomes*^*mCherry*/+^ were crossed with CD45.1^+/+^ and P14 strains to generate congenically labeled strains. The *Cd8a*^*cre*/+^ strain was crossed to *Gfi1*^fl/fl^CD45.1^+/+^ mice to generate congenically labeled mice selectively lacking GFI1 in CD8^+^ T cells. Male and female mice were used at 6–16 weeks old unless otherwise indicated. All mice were bred and maintained under specific-pathogen-free conditions at the animal facility of the University of Queensland. Mice were housed under a 12 h light/12 h dark cycle at 22 ± 2°C and 55 ± 15% humidity. All animals were handled according to the guidelines of the Australian Code for the Care and Use of Animals of the National Health and Medical Research Council of Australia. Experimental procedures were approved by the Animal Ethics Committees of the University of Queensland.

### Cell lines and virus infection

BHK-21 (CCL-10), M2-10B4 (CRL-1972) and Vero E6 (CRL-1586) cells were obtained from ATCC. All cell lines were maintained in DMEM supplemented with 10% heat-inactivated fetal calf serum (FCS), 100 U ml^−1^ penicillin and 100 μg ml^−1^ streptomycin.

LCMV^Arm^ and LCMV^c13^ were propagated and titrated on BHK-21 and Vero E6 cells, respectively. MCMV was derived from pSM3fr-MCK-2fl clone 3.3 BAC. MCMV-ie2-gp33 was kindly provided by L. Cicin-Sain^48^. MCMV strains were reconstituted by BAC transfection of M2-10B4 cells. After reconstitution, the virus was propagated on M2-10B4 cells. Virus stocks were prepared according to a previously described protocol^49^. Mice were infected intraperitoneally with 2 × 10^5^ plaque-forming units (PFU) of LCMV^Arm^ for acute viral infection. For chronic LCMV infection, mice were infected intravenously with 2 × 10^6^ PFU of LCMV^c13.^ For MCMV infection, animals were infected intraperitoneally with 2 × 10^5^ PFU of cell culture grown virus. To determine MCMV replication, tissues were homogenized and titrated on M2-10B4 cells as described previously^30^.

### Tamoxifen and FK506 treatment

Tamoxifen was dissolved in corn oil at a concentration of 20 mg ml^−1^. Infected C57BL/6 mice were treated by injecting 100 mg tamoxifen per kg body weight by intraperitoneal injection. Tamoxifen was administered once every 24 h for four consecutive days. MCMV-infected mice were treated with tamoxifen at 30 days after infection, followed by a rest period of 14–28 days before analyses. LCMV^c13^-infected mice were treated 15 days after infection, rested and then analyzed at 30 days after infection. FK506 was dissolved in 30% PEG400 and 2% Tween 80 in PBS. For FK506 treatment, 10 mg FK506 per kg body weight or vehicle was injected intraperitoneally daily from day 4–6 after virus infection.

### Cell isolation and flow cytometric analyses

Single-cell suspensions were generated by forcing tissues through 70 μm cell strainers, and red blood cells were removed using hypotonic lysis using ACK buffer (150 mM NH_4_Cl, 10 mM KHCO_3_, 0.1 mM EDTA pH 7.4). Peripheral blood samples were collected by retro-orbital bleeding, and red blood cells were lysed using ACK buffer. Lungs were perfused with approximately 5 ml PBS through the right ventricle to remove circulating blood. The lungs were placed in collagenase type IV (1 mg ml^−1^; Worthington), deoxyribonuclease I (200 μg ml^−1^; Roche) and dispase (0.4 U ml^−1^; Gibco) in complete RPMI medium (RPMI 1640 medium containing 10% heat-inactivated FCS, 1 mM l-glutamine, 100 U ml^−1^ penicillin, 100 μg ml^−1^ streptomycin and 50 μM β-mercaptoethanol) and then dissociated and homogenized using the gentleMACS Dissociator (Miltenyi Biotec) mouse lung digestion protocol setting. After dissociation, mononuclear cells were purified by gradient centrifugation using a 40–80% Percoll gradient. Cell suspensions were blocked with PBS containing 5 μg ml^−1^ anti-CD16/CD32 (2.4G2) and stained (30 min on ice) with fluorophore-conjugated antibodies or reagents in FACS buffer (PBS containing 2.5% heat-inactivated FCS and 50 mM EDTA), unless stated otherwise. Cell suspensions were incubated with fluorophore-conjugated MHCI tetramers for 30 min at 25 °C. M45-specific (H-2D^b^ restricted peptide HGIRNASFI) and M38-specific (H-2K^b^ restricted peptide SSPPMFRV) MHCI–biotin monomers were provided by the National Institutes of Health Tetramer Core Facility, and tetramers were generated using streptavidin–fluorophore conjugates. Antigen-specific CD8^+^ T cell cytokine analysis was performed by incubating single-cell suspensions with 1 µg ml^−1^ KAVYNFATM (H-2D^b^-restricted) peptide in complete RPMI medium for 1 h at 37 °C, followed by 10 μg ml^−1^ brefeldin A (Golgiplug; BD Pharmingen) addition and further 5 h incubation. For intracellular staining, surface-labeled cells were fixed using eBioscience Foxp3/Transcription Factor Staining Buffer (Thermo Fisher) and then stained for intracellular cytokines or transcription factors. Live cells were identified by exclusion staining with a fixable viability dye (BD Biosciences or BioLegend) or 7-AAD (BD Biosciences). All antibodies and staining reagents used in the study are outlined in Supplementary Table 12. Flow cytometry analysis was performed on a Cytek Aurora (Cytek Biosciences) or LSRFortessa X-20 (BD Biosciences), and analysis was performed using FlowJo software (v.10.10) (BD Biosciences).

### Adoptive CD8^+^ T cell transfer

For primary population transfer experiments, naive *Cd8a*^*cre*/+^*Gfi1*^+/+^ P14 (WT P14), *Cd8a*^*cre*/+^*Gfi1*^fl/fl^ P14 (GFI1^ΔCD8^ P14) or *Gfi1*^*tdTomato*/+^ CD8^+^ T cells were isolated from naive mice spleens using the T_N_ CD8a^+^ T Cell Isolation Kit (Miltenyi Biotec) according to the manufacturer’s instructions. Congenically labeled naive CD8^+^ P14 WT (CD45.1^+^ or CD45.1^+^CD45.2^+^) and GFI1-deficient (GFI1^ΔCD8^, CD45.1^+^ or CD45.1^+^CD45.2^+^) P14 T cells mixed at a 1:1 ratio (5 × 10^3^ cells of each type) were adoptively transferred into C57BL/6 (CD45.2^+^) recipient mice. Then, 1 day later, recipient mice were infected with the specified virus. For secondary transfer of transgenic cells, P14 T cells were first enriched from the spleen and lymph nodes of primary recipients using the CD8a^+^ T Cell Isolation Kit (Miltenyi Biotec). Cells were then stained with anti-mouse CD3ε, anti-mouse CD8a, anti-mouse CD45.1, anti-mouse CD45.2 and 7-AAD to allow discrimination of live and dead cells. Cells were then flow-cytometrically sorted on a BD FACSAria II (BD Biosciences) or Aurora CS Cell Sorter (Cytek Biosciences), and 1 × 10^4^ cells were transferred into each recipient. The secondary response and expansion capacity of GFI1^hi^ and GFI1^lo^ CD8^+^ T cells was evaluated following adoptive transfer of 1 × 10^5^ cells into the secondary host. For evaluating virus control capacity of CD8^+^ T cells, 1 × 10^5^ activated WT or GFI1^ΔCD8^ P14 T cells were transferred into *Rag2*^–/–^*Il2rγ*^–/–^ mice followed by MCMV-ie2-gp33 infection the next day. Viral titer was determined at day 14 after infection or at the time of death in liver and lungs. Unless specified otherwise, equal numbers of cells of each CD8^+^ T cell subset were injected for secondary transfer into naive or infection-matched secondary hosts.

### In vitro T cell culture

Naive WT and GFI1^ΔCD8^ P14 T cells were isolated using the Naive CD8a^+^ T Cell Isolation Kit (Miltenyi Biotec). Enriched naive congenically labeled P14 T cells were adoptively transferred into C57BL/6 (CD45.2^+^) recipients, which were infected with LCMV^c13^ 24 h later. P14 T cells were isolated from the spleen of infected animals 5 days after infection using the BD FACSAria II (BD Biosciences) or Aurora CS Cell Sorter (Cytek Biosciences). The CD8^+^ T cells were labeled with 5 μM CellTrace Violet dye (Thermo Fisher) according to the manufacturer’s recommended protocol, and 5 × 10^3^ T cells were cultured in complete RPMI 1640 medium. Complete RPMI medium was supplemented with either IL-2 (30 U ml^−1^; Thermo Fisher), IL-2 + IL-7 (10 ng ml^−1^ IL-7; Thermo Fisher) or IL-2 + CD3/CD28 beads (Dynabeads Mouse T-Activator CD3/CD28, Thermo Fisher). Dynabeads Mouse T-Activator CD3/CD28 beads were added at a 1:1 ratio to cells per well. CellTrace Violet expression and T cell expansion were quantified using Cytek Aurora (Cytek Biosciences).

### Generation of bone marrow chimeric mice

C57BL/6 recipient mice (6–10 weeks old) were lethally irradiated with two doses of 5.5 Gy (3 h apart). Bone marrow cells were isolated from *Cd8a*^*cre*/+^*Gfi1*^+/+^ or *Cd8a*^*cre*/+^*Gfi1*^fl/fl^ (CD45.1^+^) and C57BL/6 (CD45.2^+^) donor mice by flushing the femoral and tibial bones with 3 × 1 ml sterile FACS buffer to create a single-cell suspension. Red blood cells were lysed using ACK buffer and then washed twice with FACS buffer. Live cells were enumerated using Trypan blue exclusion. *Cd8a*^*cre*/+^*Gfi1*^fl/fl^ or *Cd8a*^*cre*/+^*Gfi1*^+/+^ (CD45.1^+^ or CD45.1^+^CD45.2^+^) bone marrow cells were mixed in a 1:1 ratio with C57BL/6 (CD45.2^+^) bone marrow cells, and 2–4 × 10^6^ mixed bone marrow cells were then adoptively transferred into the irradiated recipients. Chimeric mice were allowed 6–10 weeks to fully reconstitute their hematopoietic system with donor bone marrow cells before viral infection.

### Lentivirus transduction

An EOMES overexpression construct was generated by conjugating the EOMES open reading frame with an EF1a-driven enhanced green fluorescent protein (EGFP) using a T2A linker (pLV-EF1a-EGFP-T2A-EOMES). Lentiviruses were produced by the University of Queensland Viral Vector Core. CD3/CD28 bead-activated P14 T cells were spinoculated with lentiviruses carrying a control (pLV-EF1a-EGFP-T2A-Puro) or the EOMES overexpression construct. In brief, 200 µl of the lentivirus suspension was centrifuged at 3,000*g* at 32 °C for 2 h in a 48-well plate coated with RetroNectin (Takara Bio) according to the manufacturer’s instructions. Next, 5 × 10^5^ P14 T cells resuspended in complete RPMI medium supplemented with 100 ng ml^−1^ of mIL-2 was added to 400 µl per well. The cells were then centrifuged at 800*g* at 32 °C for 1.5 h. After 2 d in vitro culture, transduced CD8^+^ T cells were sorted by flow cytometry to enrich for GFP^+^-transduced P14 cells. 1–5 × 10^3^ GFP^+^P14 (CD45.1^+^ or CD45.1^+^CD45.2^+^) CD8^+^ T cells were adoptively transferred into C57BL/6 recipient mice, which were then infected with LCMV^c13^.

### RNA isolation and bulk RNA-seq

P14 CD8^+^ T cells were sorted from splenocytes isolated from mice on day 7 or day 21 after viral infection. Total RNA was extracted using RNeasy Plus Micro kit (Qiagen) according to the manufacturer’s instructions. The quality and integrity of total RNA was measured using Bioanalyzer or TapeStation systems (Agilent Technologies). Libraries were prepared using the TruSeq Stranded Total RNA Kit (Illumina) or NEBNext Single Cell/Low Input Library Prep Kit (New England Biolabs) and sequenced using a NovaSeq S1 PE100 flow cell (Illumina) or NovaSeq SP 100 flow cell (Illumina).

RNA-seq read quality was assessed, and low-quality reads were trimmed with *fastp* (v.0.22.0)^50^. Reads were mapped to the mouse genome (mm10) using STAR (v.2.7.10)^51^ and quantified with featureCounts (v.2.0.1)^52^. Read counts were normalized, and differential gene expression was quantified with DESeq2 (v.1.4.0). A log(fold change) larger than one and a false discovery rate cutoff of 5% was used to select significantly over-represented and under-represented genes. Gene set enrichment analysis was performed using *clusterProfiler* (v.4.8.3). Volcano plots and heatmaps were plotted using *EnhancedVolcano* (v.1.18.0) and *pheatmap* (v.1.0.12) packages, respectively.

### Promotor motif discovery analysis

A list of DEGs, expressed in GFI1^ΔCD8^ at day 7 after LCMV^c13^ infection (Supplementary Table 5), was used to discover the presence of the GFI1 binding motif in the promotor and enhancer regions of sequences using the *findMotifs.pl* command (HOMER v.5.1) with default parameters. The GFI1 binding motif weight matrix was downloaded from the SwissRegulon Portal or JASPAR databases.

### Bulk ATAC-seq

ATAC-seq was performed using the Omni-ATAC protocol^53^ with minor modifications. P14 CD8^+^ T cells were flow-cytometrically sorted from mice on day 3, 5 or 7 after virus infection. A total of 50,000 sorted P14 CD8^+^ T cells were lysed to extract nuclei using cell lysis buffer (10 mM Tris-HCl, 10 mM NaCl, 3 mM MgCl_2_, 0.1% Tween-20, 0.1% IGEPAL CA-630, 0.01% digitonin and 1% BSA). Nuclei were washed and resuspended in 50 μl 1× TDE1 buffer (Illumina) containing 2.5 μL TDE1 transposase (Illumina). The transposase reaction was conducted at 37 °C for 30 min with mild shaking. Library amplification and barcoding were performed with NEBNext Ultra II Q5 Master Mix (New England Biolabs) using IDT dual index primer set (Integrated DNA Technologies). PCR was conducted for 10–11 cycles. Library purification was performed with the MinElute PCR Purification Kit (Qiagen), and library size distribution was assessed using the TapeStation High Sensitivity DNA Kit (Agilent). ATAC-seq libraries were quantified before pooling and sequencing using the real-time NEBNext Library Quant Kit for Illumina (New England Biolabs). Paired-end sequencing was performed on a NovaSeq SP 100 flow cell (Illumina) with 50 cycles for each read.

Sequencing read quality was assessed, and low-quality reads were trimmed with fastp (v.0.22.0)^50^. These trimmed reads were mapped to the mouse genome (mm10) using bowtie2 (v.2.4.2) with standard parameters^54^. Picard (v.2.26.4) was used to remove PCR duplicates. The deduplicated reads were then filtered to remove mitochondrial chromosome, Y chromosome, improperly paired and non-mapping reads using samtools flags. Peak summits were called using macs2 (v.2.2.9.1) using parameters --*nomodel, --keep-dup all* and *--call-summits*. ATAC-seq library normalization was performed using the trimmed mean of M values method. Identification of differentially accessible regions (fold change of at least one and a false discovery rate of <0.05) was performed using DiffBind (v.3.12.0). Peaks were annotated using ChIPpeakAnno (v.3.36.1)^55^.

### scMultiome-seq

scmultiome-seq was performed using the 10× Single Cell Multiome ATAC + GEX analyses kit (10× Genomics). P14 CD8^+^ T cells were FAC-sorted from mice at day 7 after LCMV^c13^ infection. CD8^+^ T cells were pooled from 3–5 mice, and ~1 × 10^5^ cells were used for nuclei isolation. Cells were pelleted for 5 min at 300*g* followed by nuclei isolation by incubating cells with chilled cell lysis buffer (10 mM Tris-HCl, 10 mM NaCl, 3 mM MgCl_2_, 0.1% Tween-20, 0.1% IGEPAL CA-630, 0.01% digitonin, 1 mM dithiothreitol, 1 U μl^−1^ RNase inhibitor and 1% BSA) for 3 min. Nuclei were washed twice in 1 ml of wash buffer (10 mM Tris-HCl pH 7.4, 10 mM NaCl, 3 mM MgCl_2_, 0.1% Tween-20, 1 mM dithiothreitol, 1 U µl^−1^ RNase inhibitor and 1% BSA) by centrifuging at 500*g* for 5 min. After centrifugation, cells were resuspended in chilled Nuclei Buffer (1× Nuclei Buffer, 1 mM dithiothreitol and 1 U µl^−1^ RNase inhibitor). Nuclei were incubated in a transposition mix according to the Chromium Next GEM Single Cell Multiome ATAC + GEX user guide (protocol CG000338 Rev F). Following transposition, GEMs were generated using 10× Chip J. Sample cleanup and amplification PCR were performed as per the user guide. For the ATAC-seq library, eight PCR cycles were run, and seven PCR cycles were used for cDNA amplification. ATAC libraries were sequenced using the NovaSeq SP 100 flow cell (Illumina) with the following read protocol: 50 cycles (read 1), 8 cycles (i7 index read), 24 cycles (i5 index read) and 49 cycles (read 2). RNA libraries were sequenced on a NovaSeq SP 100 flow cell (Illumina) with the following settings: 28 cycles (read 1), 10 cycles (i7 index read), 10 cycles (i5 index read) and 90 cycles (read 2).

### Single-cell multiome data processing

RNA and ATAC raw reads from WT and GFI1^ΔCD8^ T cell samples were processed with CellRanger-arc (v.2.0.2) to map RNA transcripts and ATAC peaks to the mm10 reference genome. Seurat objects (WT and GFI1^ΔCD8^ T cells) were created using RNA matrix files using Seurat (v.5.0.3)^56^. The ATAC data were added to the Seurat object using *CreateChromatinAssay* (Seurat). Transcription start site enrichment and nucleosome signal scores were calculated using Signac (v.1.12.0)^57^. Quality control was performed by filtering cells with the following criteria: transcription start site enrichment score of >1, a nucleosome signal score of <2, between 100 and 15,000 total RNA counts, between 2,000 and 30,000 total ATAC counts and percent mitochondrial counts of <20. Cell cycle scores were assigned based on G2/M and S phase variability scores using the Seurat *CellCycleScoring* function. The Seurat object was split into RNA and ATAC objects for individual processing.

RNA gene expression unique molecular identifier count data were normalized using *SCTransform*, and principal component analysis was performed on the SCTransformed Pearson residual matrix using the *RunPCA* function in Seurat. We found the 50 nearest neighbors for each cell using the Louvain algorithm with the *FindNeighbors* function. After preprocessing, WT and GFI1^ΔCD8^ T cell RNA data were merged and integrated using the *IntegrateLayers* (RPCAIntegration) function of Seurat.

For WT and GFI1^ΔCD8^ T cell ATAC data, consensus peaks were called using the Signac *CallPeaks* function. ATAC data were processed by computing term-frequency inverse-document-frequency and running singular value decomposition using the Signac *RunTFIDF* and *RunSVD* function. Dimension reduction was performed on the ATAC dataset using latent semantic indexing (LSI) and UMAP. Then, graph-based clustering was performed on LSI components 2 to 30 by first computing a shared nearest neighbor graph using LSI low-dimensional space and then applying the Louvain algorithm using the *FindNeighbors* function followed by *FindClusters* with algorithm = 2 in Seurat. WT and GFI1^ΔCD8^ T cell ATAC data were integrated using low-dimensional cell embeddings across datasets using the Signac *IntegrateEmbeddings* function. Finally, UMAP dimensional reduction was performed using integrated LSI to visualize the integrated data.

The Seurat *FindMultiModalNeighbors* function was used to compute a joint neighbor graph that represented both the gene expression and DNA accessibility measurements using the weighted nearest neighbor methods. UMAP was performed using a joint weighted nearest neighbor map followed by unsupervised clustering using the Seurat *FindClusters* function. Integrated UMAP plots were produced using *the DimPlot* function with a Viridis color scale. Marker genes were identified by the Seurat *FindAllMarkers* function. DEGs and DARs between groups of cells were calculated using the *FindMarkers* function. DEGs and DARS were classified as an adjusted *P* value (Bonferroni-corrected) of <0.01 and absolute log_2_(fold change) of >1. Chromatin accessibility track plots were generated using the Signac *CoveragePlot* function, and gene expression was taken from normalized non-SCtransformed RNA data.

### SCENIC+ analysis

Integrated single-cell transcriptomic and single-cell chromatin accessibility data from the Seurat analysis was used to identify gene regulatory networks using the SCENIC+ (v.1.0a1) algorithm as described previously^33^. Topic modeling, dimensionality reduction, dropout imputation and differential accessibility region inference were performed using pycisTopic (v.2.0) with default parameters. A serial latent Dirichlet allocation model with collapsed Gibbs sampler (500 iterations) was used for topic modeling. Topics ranged from 2 to 500, with the final model comprising 200 topics. PycisTarget (v.1.0) was used with default settings to incorporate cisTarget and differential enrichment of motifs using bulk consensus peaks motif database. SCENIC+ was run with default parameters, and http://ensembl.org/biomart was used as the *biomaRt* host. eRegulon results were filtered based on both the correlation between gene-based regulon area under the curve and region-based regulon area under the curve with a cutoff of >0.7. Gene regulatory networks identified by SCENIC+ analysis were plotted using Cytoscape (v.3.10.0).

### Quantification and statistical analysis

Statistical analysis was performed using Prism (v.10.0) software (GraphPad). Data are shown as the mean ± s.e.m. Data distribution was assumed to be normal, but this was not formally tested.

### Reporting summary

Further information on research design is available in the Nature Portfolio Reporting Summary linked to this article.

## Online content

Any methods, additional references, Nature Portfolio reporting summaries, source data, extended data, supplementary information, acknowledgements, peer review information; details of author contributions and competing interests; and statements of data and code availability are available at 10.1038/s41590-025-02151-5.

## Supplementary information

Reporting Summary

Supplementary Table Supplementary Tables 1–12.

## Data Availability

RNA-seq and ATAC-seq data have been deposited in the Gene Expression Omnibus repository with accession number GSE271885. All other data generated or analyzed in this study are included in the paper and Supplementary Information files.

## Code availability

No custom code or algorithms were used in this study.
