# Supplementary material for: GFI1-driven transcriptional and epigenetic programs maintain CD8+ T cell stemness and persistence
Source: Nat Immunol. 2025 May 15;26(6):894–907. doi: 10.1038/s41590-025-02151-5 (PMC12133582; doi:10.1038/s41590-025-02151-5)
Supplement: Supplementary file 1 — Reporting Summary [file 41590_2025_2151_MOESM1_ESM.pdf]

nature portfolio

Corresponding author(s): Gabrielle T. Belz

Last updated by author(s): Mar 29, 2025

Reporting Summary

Nature Portfolio wishes to improve the reproducibility of the work that we publish. This form provides structure for consistency and transparency in reporting. For further information on Nature Portfolio policies, see our Editorial Policies and the Editorial Policy Checklist.

Statistics

For all statistical analyses, confirm that the following items are present in the figure legend, table legend, main text, or Methods section.

n/a	Confirmed	
☐	☒	The exact sample size (*n*) for each experimental group/condition, given as a discrete number and unit of measurement
☐	☒	A statement on whether measurements were taken from distinct samples or whether the same sample was measured repeatedly
☐	☒	The statistical test(s) used AND whether they are one- or two-sided<br>*Only common tests should be described solely by name; describe more complex techniques in the Methods section.*
☒	☐	A description of all covariates tested
☐	☒	A description of any assumptions or corrections, such as tests of normality and adjustment for multiple comparisons
☐	☒	A full description of the statistical parameters including central tendency (e.g. means) or other basic estimates (e.g. regression coefficient) AND variation (e.g. standard deviation) or associated estimates of uncertainty (e.g. confidence intervals)
☐	☒	For null hypothesis testing, the test statistic (e.g. *F*, *t*, *r*) with confidence intervals, effect sizes, degrees of freedom and *P* value noted<br>*Give P values as exact values whenever suitable.*
☒	☐	For Bayesian analysis, information on the choice of priors and Markov chain Monte Carlo settings
☒	☐	For hierarchical and complex designs, identification of the appropriate level for tests and full reporting of outcomes
☐	☒	Estimates of effect sizes (e.g. Cohen's *d*, Pearson's *r*), indicating how they were calculated

Our web collection on statistics for biologists contains articles on many of the points above.

Software and code

Policy information about availability of computer code

Data collection

Flow cytometry analysis was performed on a Cytek Aurora (Cytek Biosciences) or LSRFortessa X-20 (BD Biosciences). Bulk RNA-seq, bulk ATAC-seq and 10X single-cell multiome-seq libraries were sequenced using NovaSeq S1 PE100 flow cell (Illumina) or NovaSeq SP 100 flow cell (Illumina).

Data analysis

All flow cytometry data were analysed using FlowJo analysis software v.10.10 (BD Biosciences). Statistical analysis was performed using Prism v10.0 software (GraphPad Software).
For bulk RNA-seq, RNA-seq read quality was assessed and low quality reads trimmed with fastp v0.22.0. Reads were mapped to the mouse genome (mm10) using STAR v2.7.10 and quantified with featureCounts v2.0.1. Read counts were normalized and differential gene expression quantified with DESeq2 v1.4.0. A log-fold change larger than one and a false discovery rate cut-off of 5% was used to select significantly over- and under-represented genes. Geneset enrichment analysis was performed using clusterProfiler v4.8.3. Volcano plots and heatmaps were plotted using EnhancedVolcano v1.18.0 and pheatmap v1.0.12 packages, respectively.
Bulk ATAC-seq read quality was assessed and low quality reads trimmed with fastp v0.22.0. These trimmed reads were mapped to the mouse genome (mm10) using bowtie2 v2.4.2, with standard parameters. Picard v2.26.4 was used to remove PCR duplicates. The deduplicated reads were then filtered to remove mitochondrial chromosome, Y chromosome, improperly paired and non-mapping reads using samtools flags. Peak summits were called using macs2 v2.2.9.1 using parameter --nomodel, --keep-dup all and --call-summits. ATAC-seq library normalization was performed using the trimmed mean of M values (TMM) method. Identification of differentially accessible regions (at least a 1-fold-change and false discovery rate less than 0.05) was performed using DiffBind v3.12.0. Peaks were annotated using ChIPpeakAnno v3.36.1.
Single-cell multiome sequencing data was analysed using CellRanger-arc, Seurat v5.0.3 and Signac v1.12.0. SCENIC+ v1.0a1 was used to identify gene regulatory networks. Topic modelling, dimensionality reduction, dropout imputation and differential accessibility region inference were performed using pycisTopic v2.0 with default parameters. Serial latent dirichlet allocation model with collapsed Gibbs sampler (500 iterations) was used for topic modelling. Topics ranged from 2 to 500, with the final model comprising 200 topics. PycisTarget v1.0 was

used with default settings to incorporate cisTarget and differential enrichment of motifs using bulk consensus peaks motif database. The SCENIC+ was run with default parameters and http://ensembl.org/biomart/ was used as the biomaRt host. eRegulon results were filtered based on both the correlation between gene-based regulon area under the curve (AUC) and region-based regulon AUC with the cut off AUC > 0.7. Gene regulatory networks identified by SCENIC+ analysis were plotted using Cytoscape v3.10.0.
For Motif discovery analysis, findMotifs.pl command (HOMER v5.1) with default parameters was used. The GFI1 binding motif weight matrix was downloaded from the SwissRegulon Portal or JASPAR databases.

For manuscripts utilizing custom algorithms or software that are central to the research but not yet described in published literature, software must be made available to editors and reviewers. We strongly encourage code deposition in a community repository (e.g. GitHub). See the Nature Portfolio guidelines for submitting code & software for further information.

Data

Policy information about availability of data

All manuscripts must include a data availability statement. This statement should provide the following information, where applicable:

- Accession codes, unique identifiers, or web links for publicly available datasets
- A description of any restrictions on data availability
- For clinical datasets or third party data, please ensure that the statement adheres to our policy

RNA and ATAC sequencing data have been deposited in the Gene Expression Omnibus repository with the accession number GSE271885. All other data generated or analysed in this study are included in the published version and supplementary information files.

Research involving human participants, their data, or biological material

Policy information about studies with human participants or human data. See also policy information about sex, gender (identity/presentation), and sexual orientation and race, ethnicity and racism.

Reporting on sex and gender	N/A
Reporting on race, ethnicity, or other socially relevant groupings	N/A
Population characteristics	N/A
Recruitment	N/A
Ethics oversight	N/A

Note that full information on the approval of the study protocol must also be provided in the manuscript.

Field-specific reporting

Please select the one below that is the best fit for your research. If you are not sure, read the appropriate sections before making your selection.

☒ Life sciences ☐ Behavioural & social sciences ☐ Ecological, evolutionary & environmental sciences

For a reference copy of the document with all sections, see nature.com/documents/nr-reporting-summary-flat.pdf

Life sciences study design

All studies must disclose on these points even when the disclosure is negative.

Sample size	Animal models used in this study are well established and based on prior research conducted in our laboratories to use sufficient numbers of mice or cells in each group. For each experiment 3-5 animals were used and experiments were repeated
Data exclusions	No data points were excluded.
Replication	All experiments, except RNA and ATAC sequencing (including single-cell sequencing), were performed at least twice. The presented data were successfully replicated. Bulk RNA-seq and bulk ATAC-seq data findings were reproduced with single-cell multiomic anlaysis. All findings were reproduced
Randomization	Age- and sex-matched mice were allocated to groups based on the experimental treatment (no randomisation).
Blinding	Blinding was not performed in this study as data analysis in strictly quantitative (and not subjective) and data acquisition occurred based on fixed protocols. Thus, introduction of investigator bias is unlikely.

Reporting for specific materials, systems and methods

We require information from authors about some types of materials, experimental systems and methods used in many studies. Here, indicate whether each material, system or method listed is relevant to your study. If you are not sure if a list item applies to your research, read the appropriate section before selecting a response.

Materials & experimental systems

n/a	Involved in the study
☐	☒ Antibodies
☐	☒ Eukaryotic cell lines
☒	☐ Palaeontology and archaeology
☐	☒ Animals and other organisms
☒	☐ Clinical data
☒	☐ Dual use research of concern
☒	☐ Plants

Methods

n/a	Involved in the study
☒	☐ ChIP-seq
☐	☒ Flow cytometry
☒	☐ MRI-based neuroimaging

Antibodies

Antibodies used

BCL2-PE/Cy7 clone 10C4 Thermo Fisher catalogue# 25-6992-42, dilution used 1:100
CD107a-FITC clone ID4B BD catalogue# 553793, dilution used 1:200
CD11a -BV421clone H155-78 Thermo Fisher catalogue# 141013, dilution used 1:100
CD16/32 clone 2.4G2 BD catalogue# 553142, dilution used 1:100
CD19-BV605 clone 1D3 BD catalogue# 563148, dilution used 1:200
CD127 -PE/Cy7 clone SB-199 Thermo Fisher catalogue# 25-1273-82, dilution used 1:200
CD27-BV510 clone LG.3A10 BD catalogue# 563605, dilution used 1:200
CD3ε-BUV395 clone 145-2C11 BioLegend catalogue# 563565, dilution used 1:100
CD3ε-BUV615 clone 145-2C11 BD catalogue# 1284741, dilution used 1:100
CD4-FITC clone GK1.5 BD catalogue# 557956, dilution used 1:200
CD4-Alexa Fluor 700 clone RM4-5 Thermo Fisher catalogue# 11-0041-85, dilution used 1:200
CD44-BUV496 clone IM7 BD catalogue# 741057, dilution used 1:500
CD45-BUV805 clone 30-F11 BD catalogue# 748370, dilution used 1:200
CD45.1-APC/eFluor 780 clone A20 Thermo Fisher catalogue# 47-0453-82, dilution used 1:200
CD45.1-FITC clone A20 BD catalogue# 5533775, dilution used 1:400
CD45.2-eFluor 450 clone 104 Thermo Fisher catalogue# 48-0454-82, dilution used 1:200
CD62L-BV786 clone MEL-14 BD catalogue# 564109, dilution used 1:1000
CD8a-PerCP/Cy5.5 clone 53-6.7 BD catalogue# 551162, dilution used 1:100
CX3CR1-BV605 clone SA011F11 BioLegend catalogue# 149027, dilution used 1:500
CXCR3-BUV737 clone CXCR3-173 BD catalogue# 741895, dilution used 1:200
EOMES-PE/Cy5 clone Dan11mag Thermo Fisher catalogue# 15-4875-82, dilution used 1:100
EOMES-PE/eF610 clone Dan11mag Thermo Fisher catalogue# 61-4875-82, dilution used 1:100
FOXO1-PE clone C29H4 Cell Singnalling Technologies catalogue# 14262S, dilution used 1:50
Granzyme B-PE/Cy7 clone NGZB Thermo Fisher catalogue# 25-8898-82, dilution used 1:100
IFNg-PE clone XMG1.2 BioLegend catalogue# 505808, dilution used 1:100
Ki-67-Alexa Fluor 488 clone SolA15 BD catalogue# 53-5698-82, dilution used 1:200
Ki-67-BUV395 clone B56 BD catalogue# 564071, dilution used 1:100
KLRG1-BV711 clone 2F1 BD catalogue# 564014, dilution used 1:200
KLRG1-Super Bright 702 clone 2F1 Thermo Fisher catalogue# 67-5893-82, dilution used 1:200
LAG3-APC clone C9B7W BD catalogue# 562346, dilution used 1:200
NK1.1-BV650 clone PK136 BD catalogue# 564143, dilution used 1:500
Perforin-APC clone eBioOMAK-D Thermo Fisher catalogue# 17-9392-80, dilution used 1:100
PD1-BV421 clone J43 BD catalogue# 562584, dilution used 1:100
PD1-PE/CF594 clone J43 BD catalogue# 562523, dilution used 1:100
Sca-1-Super Bright 645 clone D7 Thermo Fisher catalogue# 64-5981-82, dilution used 1:200
T-BET-PE/Cy5 clone eBio4B10 Thermo Fisher catalogue# 15-5825-82, dilution used 1:100
T-BET-PE/Cy7 clone eBio4B10 Thermo Fisher catalogue# 25-5825-82, dilution used 1:100
TCF1-AF488 clone S33-966 BD catalogue# 567018, dilution used 1:100
TCF1-PE clone S33-966 BD catalogue# 564217, dilution used 1:100
TCF1-BV421 clone S33-966 BD catalogue# 566692, dilution used 1:100
TCRb-APC/eFluor 780 clone H57-597 Thermo Fisher catalogue# 47-5961-82, dilution used 1:200
TNFa-BV421 clone MP6-XT22 BD catalogue# 563387, dilution used 1:100
TNFa-APC clone MP6-XT22 Thermo Fisher catalogue# 17-7321-82, dilution used 1:100
TIM3-PE clone 5D12 BD catalogue#566346, dilution used 1:200
TOX-eFluor 660 clone TXRX10 Thermo Fisher catalogue# 50-6502-82, dilution used 1:100
Vb5.1/5.2-BUV395 clone MR-9 BD catalogue# 743004, dilution used 1:400
Vβ 8-PE clone F23.1 BD catalogue# 553862, dilution used 1:400
Ly108-BV421 clone 13G3 BD catalogue# 740090, dilution used 1:100
7-AAD BD catalogue# 559925, dilution used 1:50
FVS510 BD catalogue# 564406, dilution used 1:1000
FVS700 BD catalogue# 564997, dilution used 1:1000
CellEvent™ Caspase-3/7 Thermo Fisher catalogue# C10423, dilution used 1:100

Validation: All antibodies were titrated and validated using appropriate controls.

Eukaryotic cell lines

Policy information about cell lines and Sex and Gender in Research

Cell line source(s): BHK-21 (CCL-10), M2-10B4 (CRL-1972) and Vero E6 (CRL-1586) were originally obtained from ATCC.

Authentication: Not authenticated

Mycoplasma contamination: None detected.

Commonly misidentified lines (See ICLAC register): No commonly misidentified lines were used in this study.

Animals and other research organisms

Policy information about studies involving animals; ARRIVE guidelines recommended for reporting animal research, and Sex and Gender in Research

Laboratory animals: C57BL/6 (CD45.1+ or CD45.2+), Gfi1-tdTomato, Eomes-mCherry, Rag2gc-KO, Cd8a-cre, R26-cre/ERT2, Gfi1fl/fl and P14Tg. Both, male and female mice were used at 6-16 weeks old age. All mice were bred and maintained under specific pathogen-free conditions at the animal facility of The University of Queensland. Mice were housed under a 12-hours light/12-hours dark cycle at 22°C ± 2°C and 55% ± 15% humidity.

Wild animals: No wild animals were used in this project.

Reporting on sex: Both, male and female mice were used.

Field-collected samples: None

Ethics oversight: The University of Queensland ethics committee.

Note that full information on the approval of the study protocol must also be provided in the manuscript.

Plants

Seed stocks: *Report on the source of all seed stocks or other plant material used. If applicable, state the seed stock centre and catalogue number. If plant specimens were collected from the field, describe the collection location, date and sampling procedures.*

Novel plant genotypes: *Describe the methods by which all novel plant genotypes were produced. This includes those generated by transgenic approaches, gene editing, chemical/radiation-based mutagenesis and hybridization. For transgenic lines, describe the transformation method, the number of independent lines analyzed and the generation upon which experiments were performed. For gene-edited lines, describe the editor used, the endogenous sequence targeted for editing, the targeting guide RNA sequence (if applicable) and how the editor was applied.*

Authentication: *Describe any authentication procedures for each seed stock used or novel genotype generated. Describe any experiments used to assess the effect of a mutation and, where applicable, how potential secondary effects (e.g. second site T-DNA insertions, mosiacism, off-target gene editing) were examined.*

Flow Cytometry

Plots

Confirm that:

☒ The axis labels state the marker and fluorochrome used (e.g. CD4-FITC).

☒ The axis scales are clearly visible. Include numbers along axes only for bottom left plot of group (a 'group' is an analysis of identical markers).

☒ All plots are contour plots with outliers or pseudocolor plots.

☒ A numerical value for number of cells or percentage (with statistics) is provided.

Methodology

Sample preparation: Single cell suspensions were generated by forcing tissues through 70 m cell strainers, and red blood cells (RBC) were removed using hypotonic lysis using ACK buffer. Cell suspensions were blocked with PBS containing 5ug/ml anti-CD16/CD32 and stained (30 min on ice) with fluorophore-conjugated antibodies or reagents in FACS buffer. For intracellular staining, surface-labelled cells were fixed using eBioscience Foxp3/Transcription Factor Staining Buffer (Thermo Fisher) then stained

for intracellular cytokines or transcription factors. Live cells were identified by exclusion staining with a fixable viability dye (BD Biosciences or BioLegend) or 7-AAD (BD Biosciences).

Instrument

Flow cytometry analysis was performed on a Cytek Aurora (Cytek Biosciences) or LSRFortessa X-20 (BD Biosciences). Cell Sorting was performed using BD FACS aria II (BD Biosciences).

Software

All flow cytometry data were analysed using FlowJo analysis software v.10.10 (BD Biosciences). Statistical analysis was performed using Prism v10.0 software (GraphPad Software).

Cell population abundance

Cell populations analysed ranged from 50-1000000 cells/mouse.

Gating strategy

1. FSC-A/SSC-A were used to select lymphocyte populations.
2. FSC-A/FSC-H were used to select singlets.
3. FSC-A/viability were used to identify live cells.
4. CD3/CD8 were used to select CD8+ T cells.
5. CD45.1/CD45.2 were used to identify P14 T cells.

☒ Tick this box to confirm that a figure exemplifying the gating strategy is provided in the Supplementary Information.
